# Bis[tris­(diiso­butyl­dithio­carbamato)-μ_3_-sulfido-tri-μ_2_-di­sulfido-trimolybdenum(IV)] sulfide tetra­hydro­furan monosolvate

**DOI:** 10.1107/S2056989024002949

**Published:** 2024-04-18

**Authors:** Addison Fraker, James P. Donahue, Alex McSkimming

**Affiliations:** a Department of Chemistry, Tulane University, 6400 Freret Street, New Orleans, Louisiana 70118-5698, USA; Harvard University, USA

**Keywords:** molybdenum sulfide cluster, di­thio­carbamate ligand, μ_6_-sulfide ligand, crystal structure

## Abstract

The title compound, [Mo_3_(C_9_H_18_NS_2_)_3_(S_2_)_3_S]_2_S, crystallizes on a general position in the monoclinic space group *P*2_1_/*n* (No. 14). The cationic [Mo_3_S_7_(S_2_CN^*i*^Bu_2_)_3_]^+^ fragments are joined by a mono­sulfide dianion that forms close S⋯S contacts to each of the di­sulfide ligands on the side of the Mo_3_ plane opposite the μ_3_^2−^ ligand. The two Mo_3_ planes are inclined at an angle of 40.637 (15)°, which gives the assembly an open clamshell-like appearance. One μ_6_-S^2−^⋯S_2_^2−^ contact, at 2.4849 (14) Å, is appreciably shorter than the remaining five, which are in the range 2.7252 (13)–2.8077 (14) Å.

## Abstract 2

The structure of [Mo_3_S_7_(S_2_CN^*i*^Bu_2_)_3_]_2_(μ_6_-S) features a μ_6_S_2_^2−^ anion asymmetrically wedged between two [Mo_3_S_7_(S_2_CN^*i*^Bu_2_)_3_]^+^ cations with close μ_6_-S^2−^⋯S_2_^2−^ contacts that indicate significant covalency to the inter­actions.

## 1. Chemical context

Triangular molybdenum sulfide clusters of the form [Mo_3_S_7_(S_2_CN*R*_2_)_3_]^+^I^−^ (*R* = alkyl group) function as precatalysts for an H_2_ evolving system under both photolytic and electrolytic conditions with H_2_O serving as source of protons (Fontenot *et al.*, 2019 ▸). In the photolysis system, rapid mass spectrometry assays in the first moments of irradiation reveal the loss of atomic sulfur from the bridging S_2_^2−^ ligands to form mono­sulfido bridges and an [Mo_3_S_4_]^4+^ core prior to the onset of H_2_ evolution. In a bulk electrolysis of [Mo_3_S_7_(S_2_CN^*i*^Bu_2_)_3_]^+^·I^−^ in the presence of H_2_O, the Faradaic efficiency is observed to be only about 37%. Because the same system and set of conditions reduced methyl viologen with much higher Faradaic efficiency, it is probable the the extruded elemental sulfur is competing for reducing equivalents. 
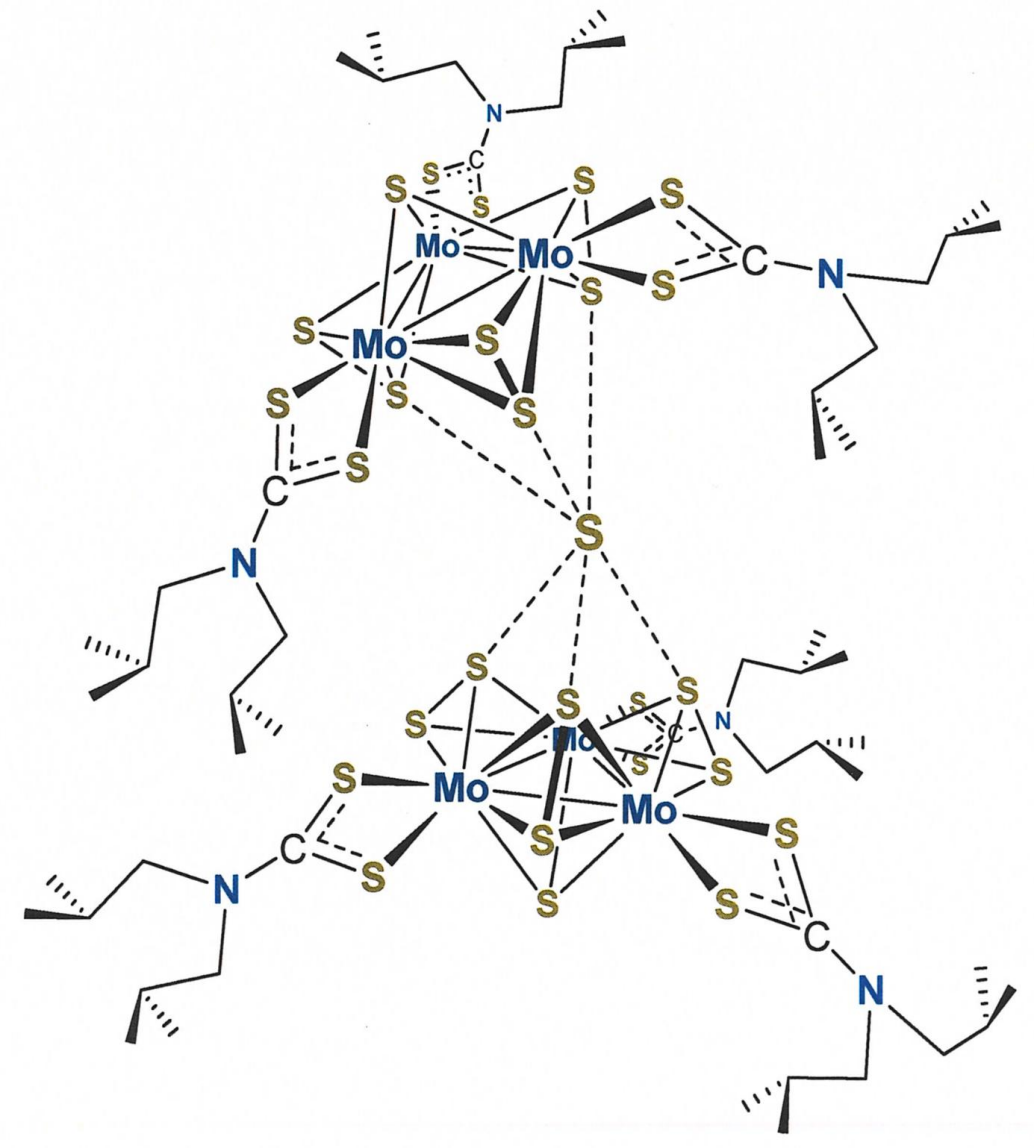


As a means of developing further insight into this system, we undertook a preparative scale reduction of [Mo_3_S_7_(S_2_CN^*i*^Bu_2_)_3_]^+^·I^−^ using the prototypical outer-sphere reductant Cp_2_Co. While the initial reaction was marked by a darkening in color, the work-up and subsequent crystallization identified yellow [Mo_3_S_7_(S_2_CN^*i*^Bu_2_)_3_]_2_(μ_6_-S) as the dominant isolable species. The presence of the sulfido counter-anion, which forms close S⋯S contacts with the axial S atoms of the bridg­ing di­sulfide ligands of two different [Mo_3_S_7_(S_2_CN^*i*^Bu_2_)_3_]^+^ clusters, confirms the diversion of electrons to free S^0^ in competition with H^+^ reduction in the bulk electrolysis. In this article, we detail the structural features of [Mo_3_S_7_(S_2_CN^*i*^Bu_2_)_3_]_2_(μ_6_-S), (I).

## 2. Structural commentary

**Figure 1:**
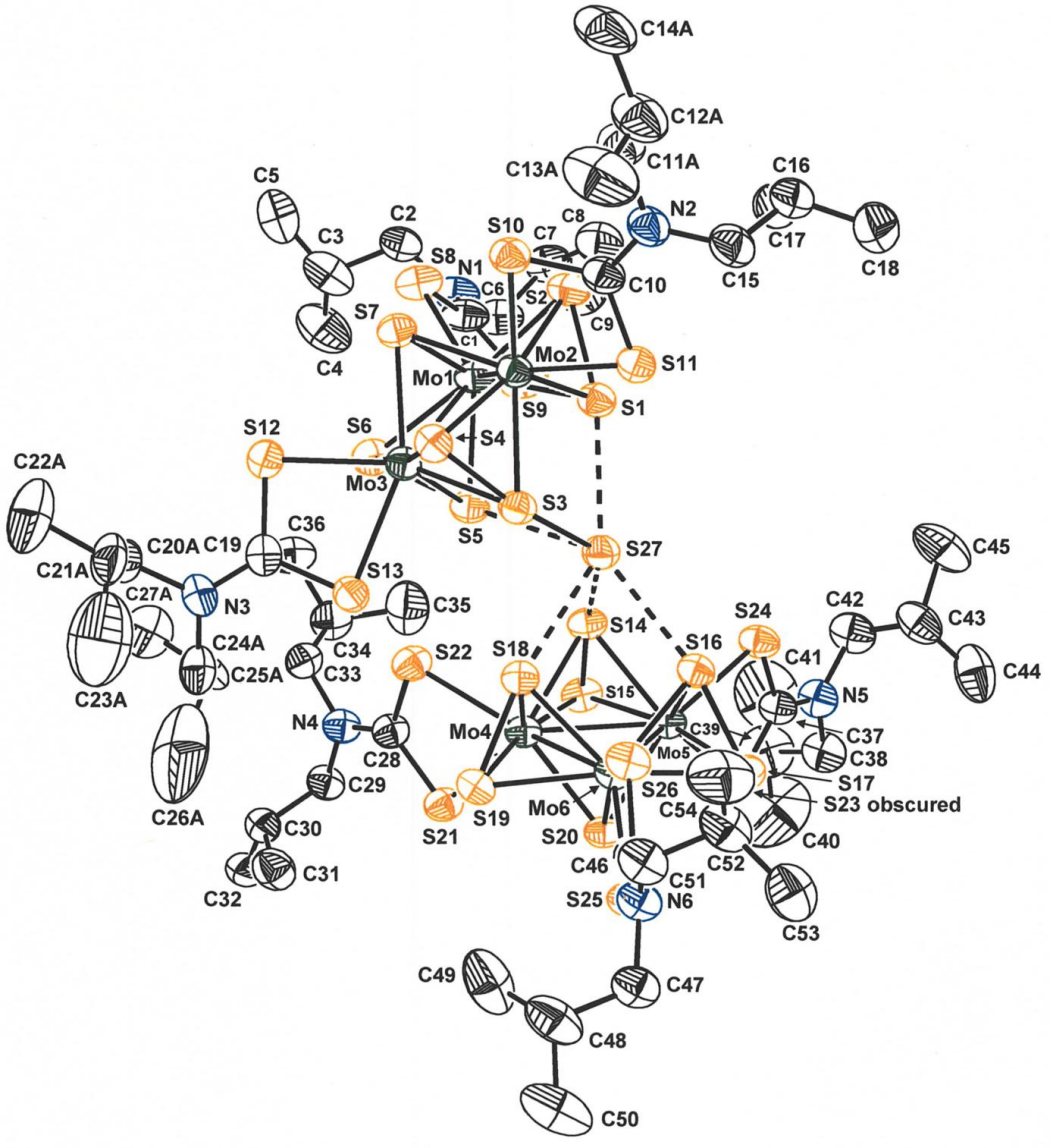
Displacement ellipsoid plot (50% probability level) of [Mo_3_S_7_(S_2_CN^*i*^Bu_2_)_3_]_2_(μ_6_-S) with complete atom labeling. For greater clarity, all H atoms and one of the two disordered parts of each disordered isobutyl group are removed.

The [Mo_3_S_7_(S_2_CN^*i*^Bu_2_)_3_]_2_(μ_6_-S) structure comprises two [Mo_3_S_7_(S_2_CN^*i*^Bu_2_)_3_]^+^ cations between which is ensconced an S^2−^ counter-anion (S27). The asymmetric joining of the two Mo_3_ clusters, as if by a hinge at S27, produces a half-opened clamshell-like appearance to the compound (Fig. 1 ▸). The angle at which these two Mo_3_ planes are disposed is 40.637 (15)° with a distance of 6.88 Å between the centroids of the two Mo_3_ triangles.

**Figure 2:**
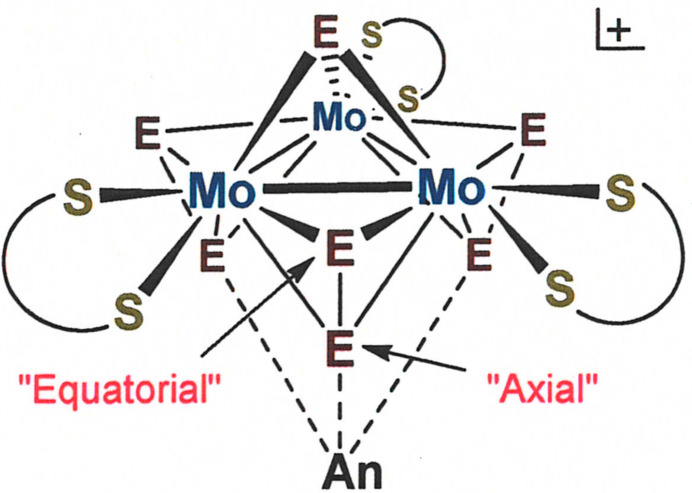
Illustration of the structural distinction between axial and equatorial sulfur atoms of the μ-S_2_^2−^ ligands in [Mo_3_S_7_(S_2_CN*R*_2_)_3_]^+^ structures, with anion position in proximity to the axial S atoms.

A general observation in the structures of [Mo_3_ *E*_7_(S_2_CN*R*_2_)_3_]^+^ (*E* = S or Se; *R* = alkyl group) complexes is that soft monoatomic counter-anions situate themselves at the ‘underside’ of the cluster cation opposite to the unique μ_3_-*E* ligand and in close proximity to the ‘axial’ chalcogen atom of the bridging dichalcogenide (Fig. 2 ▸) (Zimmermann *et al.*, 1991 ▸; Fedin *et al.*, 1992 ▸; Il’inchuk *et al.*, 2002 ▸; Lu *et al.*, 1993 ▸). These anion⋯*E*_ax_ contacts are typically less that the sum of the van der Waals radii, a fact attributed to an electrophilic character of the *E*_ax_ atom and the felicitous nature of the ‘soft–soft’ *E*_ax_⋯anion inter­action. In [Mo_3_S_7_(S_2_CN^*i*^Bu_2_)_3_]_2_(μ_6_-S), the S27⋯S_ax_ inter­atomic distances partition into two sets: the S27—S3 distance at 2.4849 (14) Å and the remaining five, which are in the range 2.7252 (13)–2.8077 (14) Å, all of which are substanti­ally less than twice the crystallographic radius for sulfur (3.6 Å; Batsanov, 2001 ▸) and therefore indicative of appreciable covalency to the inter­actions. The markedly stronger inter­action of S27 with the S3—S4 di­sulfide ligand is manifested in the S3—S4 distance being significantly longer [2.2414 (13) Å] than the remaining S—S distances in the μ-S_2_^2−^ ligands, which range from 2.0671 (13)–2.1198 (13) Å and average as 2.0857 (6) Å. This comparative elongation of the S3—S4 bond length is consistent with the proposal, as advanced in a review of the structural chemistry of [*M*_3_ *X*_7_]^4+^ and [*M*_3_ *X*_4_]^4+^ (*M* = Mo, W; *X* = O, S, Se) clusters (Virovets & Podberezskaya, 1993 ▸), that the sulfide counter-anion (S27) infuses electron density into the S3—S4 σ* orbital by overlap with one of its electron lone pairs.

**Figure 3:**
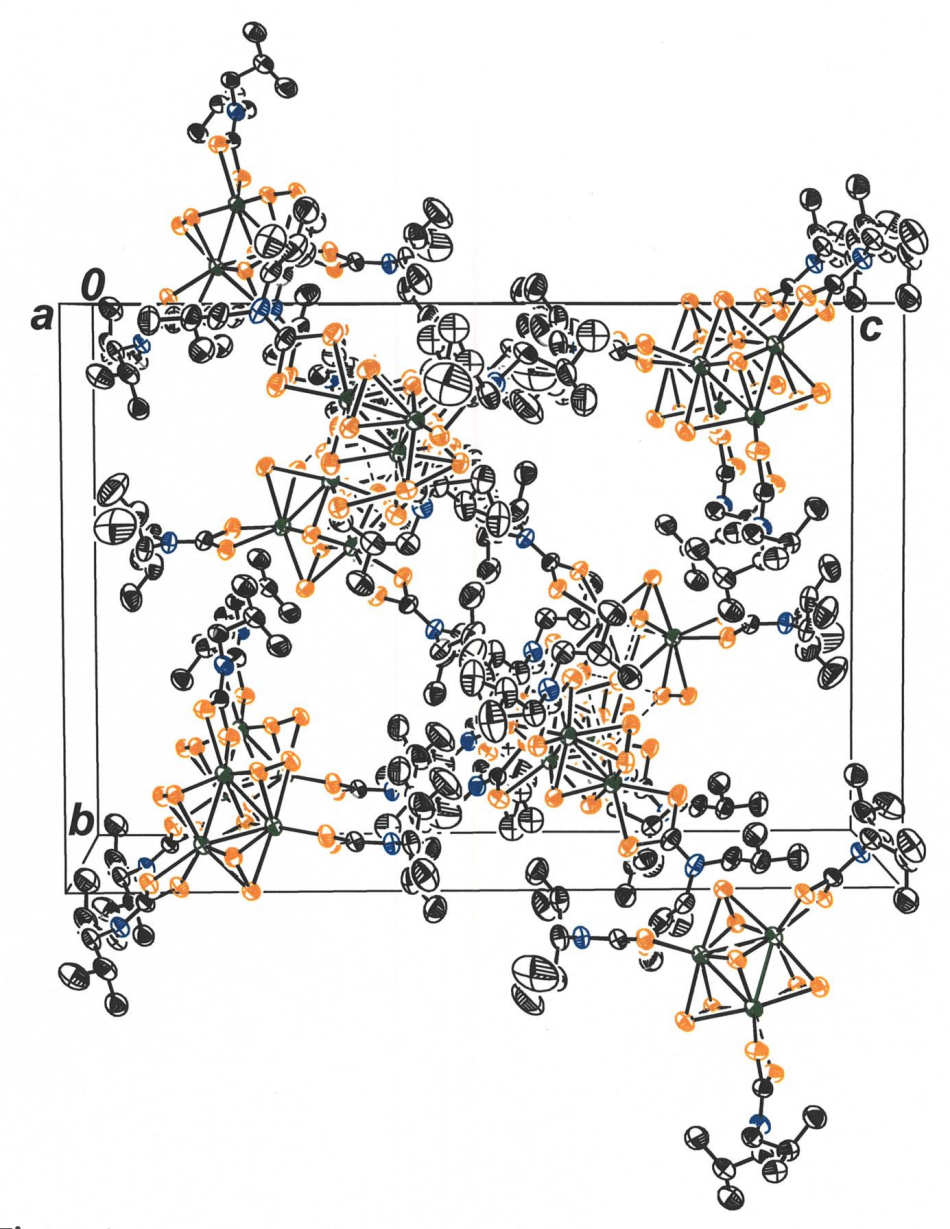
Packing arrangement for [Mo_3_S_7_(S_2_CN^*i*^Bu_2_)_3_]_2_(μ_6_-S) viewed down the *a* axis of the unit cell. Displacement ellipsoids are presented at the 50% probability level, and all H atoms are omitted for clarity.

The packing arrangement for [Mo_3_S_7_(S_2_CN^*i*^Bu_2_)_3_]_2_(μ_6_-S) places the assembly into columnar stacks along the *a* axis of the cell (Fig. 3 ▸). The *iso*butyl substituents of the ^*i*^Bu_2_NCS_2_^−^ ligands project into the spacings between these columns and likely play a decisive role in guiding the formation of this pattern by virtue of favorable dispersion-type attractive forces.

## 3. Database survey

The first reported observation of the [Mo_3_ *E*_7_(S_2_CN*R*_2_)_3_]_2_(μ_6_-*E*) (*E* = S or Se) structure type was a serendipitous formation of [Mo_3_S_7_(S_2_CNEt_2_)_3_]_2_(μ_6_-S) by substitution of the oxyquinolate (oxq) ligands in [Mo_3_S_7_(oxq)_3_]^+^ with a slight excess of Na^+^Et_2_NCS_2_^−^ in wet DMSO, the presumed source of the bridging S^2−^ ligand being the excess Et_2_NCS_2_^−^ anion *via* hydrolysis (Meienberger *et al.*, 1993 ▸). Here, the assembly crystallized in *Aba*2 (No. 41) upon a crystallographic *C*_2_ axis that was coincident with the μ_6_-S^2−^ ligand. The angle formed by the two Mo_3_ planes was 33.37° in [Mo_3_S_7_(S_2_CNEt_2_)_3_]_2_(μ_6_-S), somewhat smaller than the analogous value in [Mo_3_S_7_(S_2_CN^*i*^Bu_2_)_3_]_2_(μ_6_-S), but the Mo_3_⋯Mo_3_ centroid-to-centroid distance was 7.00 Å, slightly greater than the 6.88 Å assessed for [Mo_3_S_7_(S_2_CN^*i*^Bu_2_)_3_]_2_(μ_6_-S). Notably, the μ_6_-S^2−^⋯S_ax_ distances spanned a much more narrow range of 2.70 (1)–2.72 (1) Å than seen in [Mo_3_S_7_(S_2_CN^*i*^Bu_2_)_3_]_2_(μ_6_-S), possibly because the latter’s more sterically encumbering *iso*butyl groups have hindered close, symmetric approach to the S^2−^ bridge.

Another structure of the type with an all selenium inorganic core, [Mo_3_Se_7_(S_2_CNEt_2_)_3_]_2_(μ_6_-Se), was obtained by the oxidative addition of Et_2_NC(S)S-SC(S)NEt_2_ and Se^0^ to Mo(CO)_6_ and crystallized as an isomorph of [Mo_3_S_7_(S_2_CNEt_2_)_3_]_2_(μ_6_-S) with a similar unit cell in the same space group (Almond, *et al.*, 2000 ▸). Although larger in magnitude than the corresponding values in [Mo_3_S_7_(S_2_CNEt_2_)_3_]_2_(μ_6_-S), the spread in Se_ax_⋯μ_6_-Se^2−^ inter­atomic distances was still narrow compared to the range of analogous values in [Mo_3_S_7_(S_2_CN^*i*^Bu_2_)_3_]_2_(μ_6_-S). A pseudopolymorph of [Mo_3_Se_7_(S_2_CNEt_2_)_3_]_2_(μ_6_-Se) with inter­stitial 1,2-di­chloro­benzene revealed a similar range in Se_ax_⋯μ_6_-Se distances as seen for the structure without solvent (Brakefield *et al.*, 2020 ▸). The tungsten analogue, [W_3_Se_7_(S_2_CNEt_2_)_3_]_2_(μ_6_-Se), prepared similarly from W(CO)_6_ (Almond *et al.*, 2000 ▸), has also been described and is the only other example of the structure type.

## 4. Synthesis and crystallization

A solution of [Mo_3_S_7_(S_2_CN^*i*^Bu_2_)_3_]I (0.049 g, 0.0039 mmol) in tetra­hydro­furan (THF) was cooled to 195 K in the cold well of a glove-box. Upon cooling, a solution of cobaltocene in THF (0.0183 g, 0.0968 mol) was added dropwise to the stirring solution. This reaction mixture was stirred at 243 K for 30 min and then was removed from the cold well and warmed to room temperature with continued stirring. Upon attaining room temperature, the solution was filtered through Celite, and the volatiles were removed under reduced pressure. The oily residue was then dissolved in 20% THF in hexa­nes and passed through a 3 cm pad of silica in a glass pipette. All volatiles were then removed under reduced pressure to yield a dark-orange–brown oil. Crystals suitable for X-ray diffraction were grown by layering a concentrated THF solution with hexa­nes and maintaining the layered mixture at 243 K. Yield: 70%. ^1^H NMR (300 MHz; δ, ppm in CDCl_3_): 3.59 (*dd*, *J* = 24 Hz, 7.5 Hz, 2H, C*H*_2_), 2.22 (*m*, 1H, C*H*), 0.95 (*d*, *J* = 6.6 Hz, 6H, C*H*_3_).

## 5. Refinement

Crystal data, data collection and structure refinement details are summarized in Table 1 ▸. An initial solution for [Mo_3_S_7_(S_2_CN^*i*^Bu_2_)_3_]_2_(μ_6_-S) was obtained by direct methods and revealed the positions of most of the non-H atoms except for some peripheral C atoms of the isobutyl groups. Subsequent cycles of least-squares refinement revealed several isobutyl groups that suffered a static disorder over two positions. This disorder was treated with a split atom model that attained a best fit distribution in each case. All non-H atoms were refined anisotropically, but the disordered C atoms were treated with SIMU and RIGU restraints. All H atoms were refined isotropically as riding atoms with displacement parameters 1.2–1.5 times those of the C atoms to which they were attached. In the final difference maps, two positions occupied by disordered solvent mol­ecules were identified. These severely disordered solvent mol­ecules, which presented an electron density attributable to 367 electrons in a solvent-accessible volume of 1692 Å^3^ per unit cell, have been masked using the SQUEEZE routine (Spek, 2015 ▸) in *PLATON* (Spek, 2020 ▸).

## Supplementary Material

Crystal structure: contains datablock(s) I, global. DOI: 10.1107/S2056989024002949/oi2005sup1.cif

Structure factors: contains datablock(s) I. DOI: 10.1107/S2056989024002949/oi2005Isup3.hkl

CCDC reference: 2345828

Additional supporting information:  crystallographic information; 3D view; checkCIF report

## Figures and Tables

**Table 1:** Experimental details

Crystal data	
Chemical formula	[Mo_3_(C_9_H_18_NS_2_)_3_(S_2_)_3_S]_2_S
*M*_r_	2282.72
Crystal system, space group	Monoclinic, *P*2_1_/*n*
Temperature (K)	150
*a*, *b*, *c* (Å)	16.1699 (7), 21.1139 (10), 30.0046 (14)
β (°)	91.576 (2)
*V* (Å^3^)	10240.0 (8)
*Z*	4
Radiation type	Cu *K*α
μ (mm^−1^)	11.24
Crystal size (mm)	0.36 × 0.27 × 0.12

Data collection	
Diffractometer	Bruker D8 QUEST PHOTON 3
Absorption correction	Multi-scan (*SADABS*; Krause *et al.*, 2015 ▸)
*T*_min_, *T*_max_	0.120, 0.355
No. of measured, independent and observed [*I* > 2σ(*I*)] reflections	332865, 20942, 18768
*R*_int_	0.054
(sin θ/λ)_max_ (Å^−1^)	0.627

Refinement	
*R*[*F* ^2^ > 2σ(*F* ^2^)], *wR*(*F* ^2^), *S*	0.038, 0.089, 1.15
No. of reflections	20942
No. of parameters	967
No. of restraints	492
H-atom treatment	H-atom parameters constrained
Δρ_max_, Δρ_min_ (e Å^−3^)	1.55, −0.67

Computer programs: *APEX4* and *SAINT* (Bruker, 2021 ▸), *SHELXT* (Sheldrick, 2015*a* ▸), *SHELXL2018* (Sheldrick, 2015*b* ▸) and *SHELXTL* (Sheldrick, 2008 ▸).

## supplementary crystallographic information

### Crystal data

**Table:**

[Mo_3_(C_9_H_18_NS_2_)_3_(S_2_)_3_S]_2_S	*F*(000) = 4632
*M**_r_* = 2282.72	*D*_x_ = 1.481 Mg m^−^^3^
Monoclinic, *P*2_1_/*n*	Cu *K*α radiation, λ = 1.54178 Å
*a* = 16.1699 (7) Å	Cell parameters from 9883 reflections
*b* = 21.1139 (10) Å	θ = 5.0–74.3°
*c* = 30.0046 (14) Å	µ = 11.24 mm^−^^1^
β = 91.576 (2)°	*T* = 150 K
*V* = 10240.0 (8) Å^3^	Block, yellow
*Z* = 4	0.36 × 0.27 × 0.12 mm

### Data collection

**Table:**

Bruker D8 QUEST PHOTON 3 diffractometer	20942 independent reflections
Radiation source: INCOATEC IµS micro-focus source	18768 reflections with *I* > 2σ(*I*)
Mirror monochromator	*R*_int_ = 0.054
Detector resolution: 7.3910 pixels mm^-1^	θ_max_ = 75.1°, θ_min_ = 2.6°
φ and ω scans	*h* = −20→20
Absorption correction: multi-scan (*SADABS*; Krause *et al.*, 2015)	*k* = −26→26
*T*_min_ = 0.120, *T*_max_ = 0.355	*l* = −37→37
332865 measured reflections	

### Refinement

**Table:**

Refinement on *F*^2^	Primary atom site location: dual
Least-squares matrix: full	Secondary atom site location: difference Fourier map
*R*[*F*^2^ > 2σ(*F*^2^)] = 0.038	Hydrogen site location: inferred from neighbouring sites
*wR*(*F*^2^) = 0.089	H-atom parameters constrained
*S* = 1.15	*w* = 1/[σ^2^(*F*_o_^2^) + (0.0247*P*)^2^ + 22.8874*P*] where *P* = (*F*_o_^2^ + 2*F*_c_^2^)/3
20942 reflections	(Δ/σ)_max_ = 0.034
967 parameters	Δρ_max_ = 1.55 e Å^−^^3^
492 restraints	Δρ_min_ = −0.67 e Å^−^^3^

### Special details

**Table:**

Geometry. All esds (except the esd in the dihedral angle between two l.s. planes) are estimated using the full covariance matrix. The cell esds are taken into account individually in the estimation of esds in distances, angles and torsion angles; correlations between esds in cell parameters are only used when they are defined by crystal symmetry. An approximate (isotropic) treatment of cell esds is used for estimating esds involving l.s. planes.

### Fractional atomic coordinates and isotropic or equivalent isotropic displacement parameters (Å^2^)

**Table:**

	*x*	*y*	*z*	*U*_iso_*/*U*_eq_	Occ. (<1)
Mo1	0.28182 (2)	0.15773 (2)	0.34013 (2)	0.04249 (7)	
Mo2	0.22016 (2)	0.24608 (2)	0.39629 (2)	0.03764 (7)	
Mo3	0.36754 (2)	0.19093 (2)	0.41581 (2)	0.03837 (7)	
Mo4	0.63970 (2)	0.31015 (2)	0.31405 (2)	0.03863 (7)	
Mo5	0.57805 (2)	0.39460 (2)	0.25385 (2)	0.03791 (7)	
Mo6	0.60329 (2)	0.42944 (2)	0.34021 (2)	0.03703 (7)	
S1	0.24484 (6)	0.26354 (5)	0.31861 (3)	0.0452 (2)	
S2	0.14289 (6)	0.20676 (5)	0.32864 (3)	0.0487 (2)	
S3	0.34878 (5)	0.30402 (4)	0.40570 (3)	0.04282 (19)	
S4	0.29662 (6)	0.26176 (4)	0.46743 (3)	0.04189 (19)	
S5	0.41906 (6)	0.20052 (5)	0.34182 (3)	0.0439 (2)	
S6	0.41496 (6)	0.10737 (5)	0.36342 (4)	0.0505 (2)	
S7	0.23835 (6)	0.13682 (5)	0.41369 (3)	0.04361 (19)	
S8	0.22652 (7)	0.05089 (5)	0.32218 (3)	0.0530 (2)	
S9	0.30170 (7)	0.13312 (6)	0.25885 (4)	0.0592 (3)	
S10	0.08804 (6)	0.24067 (5)	0.43706 (3)	0.0459 (2)	
S11	0.14740 (6)	0.35163 (5)	0.39361 (3)	0.0466 (2)	
S12	0.40548 (6)	0.11981 (5)	0.47965 (4)	0.0524 (2)	
S13	0.51429 (6)	0.21189 (5)	0.44249 (3)	0.04316 (19)	
S14	0.51394 (6)	0.29511 (4)	0.27074 (3)	0.04288 (19)	
S15	0.62047 (6)	0.28593 (5)	0.23350 (3)	0.0467 (2)	
S16	0.47316 (5)	0.43703 (4)	0.30051 (3)	0.04092 (18)	
S17	0.55852 (6)	0.50476 (4)	0.28115 (3)	0.04299 (19)	
S18	0.54359 (6)	0.33574 (4)	0.37101 (3)	0.04165 (19)	
S19	0.66926 (6)	0.34979 (5)	0.39048 (3)	0.0446 (2)	
S20	0.71045 (5)	0.40274 (4)	0.29038 (3)	0.04075 (18)	
S21	0.77656 (6)	0.25674 (5)	0.31098 (4)	0.0493 (2)	
S22	0.62651 (6)	0.19647 (5)	0.33694 (4)	0.0494 (2)	
S23	0.64712 (6)	0.43142 (5)	0.18538 (3)	0.0452 (2)	
S24	0.47426 (6)	0.40163 (5)	0.18995 (3)	0.0482 (2)	
S25	0.69759 (6)	0.51203 (5)	0.37118 (3)	0.0464 (2)	
S26	0.53443 (6)	0.48741 (5)	0.40251 (3)	0.0466 (2)	
S27	0.39203 (6)	0.33071 (5)	0.32913 (3)	0.0463 (2)	
N1	0.2599 (2)	0.01216 (19)	0.23953 (12)	0.0563 (9)	
N2	−0.0071 (2)	0.34429 (18)	0.42525 (12)	0.0509 (8)	
N3	0.5663 (2)	0.12796 (16)	0.50435 (11)	0.0478 (7)	
N4	0.7610 (2)	0.12970 (16)	0.31613 (11)	0.0460 (7)	
N5	0.5473 (2)	0.42294 (18)	0.11238 (11)	0.0517 (8)	
N6	0.6341 (2)	0.57425 (16)	0.44117 (11)	0.0475 (7)	
C1	0.2620 (3)	0.0586 (2)	0.26886 (14)	0.0543 (10)	
C2	0.2326 (3)	−0.0514 (2)	0.25258 (14)	0.0527 (10)	
H2A	0.217065	−0.075287	0.225235	0.063*	
H2B	0.182288	−0.047016	0.270375	0.063*	
C3	0.2961 (3)	−0.0902 (3)	0.2795 (2)	0.0807 (17)	
H3	0.310669	−0.065912	0.307209	0.097*	
C4	0.3745 (4)	−0.1003 (4)	0.2546 (3)	0.107 (2)	
H4A	0.410117	−0.130262	0.271029	0.161*	
H4B	0.403502	−0.059789	0.251624	0.161*	
H4C	0.360986	−0.117325	0.224899	0.161*	
C5	0.2572 (4)	−0.1518 (3)	0.2932 (2)	0.0881 (18)	
H5A	0.211013	−0.143168	0.312784	0.132*	
H5B	0.298588	−0.177840	0.309208	0.132*	
H5C	0.236844	−0.174576	0.266662	0.132*	
C6	0.2842 (3)	0.0214 (3)	0.19272 (15)	0.0651 (13)	
H6A	0.321450	−0.013467	0.184158	0.078*	
H6B	0.315328	0.061625	0.190457	0.078*	
C7	0.2093 (3)	0.0233 (3)	0.15996 (16)	0.0677 (13)	
H7	0.178443	−0.017497	0.162657	0.081*	
C8	0.1505 (4)	0.0773 (3)	0.1702 (2)	0.0807 (15)	
H8A	0.179598	0.117804	0.167650	0.121*	
H8B	0.130658	0.072535	0.200612	0.121*	
H8C	0.103313	0.076383	0.149017	0.121*	
C9	0.2409 (5)	0.0281 (3)	0.11240 (18)	0.0927 (19)	
H9A	0.193806	0.028368	0.091147	0.139*	
H9B	0.276474	−0.008322	0.106332	0.139*	
H9C	0.272693	0.067296	0.109297	0.139*	
C10	0.0657 (2)	0.3159 (2)	0.41943 (13)	0.0467 (9)	
C11A	−0.0783 (8)	0.3097 (7)	0.4429 (3)	0.063 (3)	0.746 (8)
H11A	−0.058514	0.269681	0.456598	0.075*	0.746 (8)
H11B	−0.116824	0.298766	0.417830	0.075*	0.746 (8)
C12A	−0.1251 (4)	0.3477 (4)	0.4775 (2)	0.078 (2)	0.746 (8)
H12A	−0.149636	0.386074	0.462720	0.093*	0.746 (8)
C13A	−0.0681 (9)	0.3690 (9)	0.5160 (5)	0.116 (6)	0.746 (8)
H13A	−0.099958	0.393110	0.537464	0.174*	0.746 (8)
H13B	−0.043637	0.331715	0.530701	0.174*	0.746 (8)
H13C	−0.023952	0.395716	0.504460	0.174*	0.746 (8)
C14A	−0.1949 (4)	0.3061 (5)	0.4941 (3)	0.097 (3)	0.746 (8)
H14A	−0.226026	0.329373	0.516379	0.146*	0.746 (8)
H14B	−0.231995	0.294739	0.468958	0.146*	0.746 (8)
H14C	−0.171606	0.267555	0.507542	0.146*	0.746 (8)
C11B	−0.074 (2)	0.309 (2)	0.4472 (8)	0.061 (7)	0.254 (8)
H11C	−0.075433	0.266124	0.433928	0.073*	0.254 (8)
H11D	−0.126563	0.329961	0.438644	0.073*	0.254 (8)
C12B	−0.0727 (12)	0.3006 (8)	0.4983 (6)	0.065 (5)	0.254 (8)
H12B	−0.015647	0.287520	0.508124	0.079*	0.254 (8)
C13B	−0.091 (2)	0.3643 (13)	0.5197 (13)	0.071 (7)	0.254 (8)
H13D	−0.051440	0.395898	0.509358	0.106*	0.254 (8)
H13E	−0.147271	0.377504	0.511416	0.106*	0.254 (8)
H13F	−0.085519	0.360359	0.552238	0.106*	0.254 (8)
C14B	−0.1326 (17)	0.2501 (12)	0.5136 (8)	0.094 (8)	0.254 (8)
H14D	−0.119560	0.209714	0.499327	0.141*	0.254 (8)
H14E	−0.127539	0.245467	0.546041	0.141*	0.254 (8)
H14F	−0.189290	0.262613	0.505219	0.141*	0.254 (8)
C15	−0.0234 (3)	0.4080 (2)	0.40781 (16)	0.0593 (11)	
H15A	0.029939	0.427814	0.400247	0.071*	
H15B	−0.048039	0.433821	0.431560	0.071*	
C16	−0.0815 (3)	0.4096 (3)	0.36630 (18)	0.0647 (12)	
H16	−0.137341	0.394547	0.375071	0.078*	
C17	−0.0519 (3)	0.3679 (3)	0.32920 (18)	0.0722 (14)	
H17A	−0.090245	0.371309	0.303445	0.108*	
H17B	−0.049600	0.323873	0.339418	0.108*	
H17C	0.003383	0.381532	0.320612	0.108*	
C18	−0.0890 (4)	0.4786 (3)	0.3512 (2)	0.0830 (17)	
H18A	−0.034711	0.494148	0.342432	0.124*	
H18B	−0.109108	0.504487	0.375761	0.124*	
H18C	−0.128069	0.481425	0.325710	0.124*	
C19	0.5038 (3)	0.15058 (19)	0.47930 (13)	0.0455 (8)	
C20A	0.5540 (16)	0.0812 (5)	0.5400 (3)	0.056 (3)	0.750 (10)
H20A	0.606215	0.057712	0.545571	0.067*	0.750 (10)
H20B	0.511224	0.050426	0.529994	0.067*	0.750 (10)
C21A	0.5272 (5)	0.1125 (4)	0.5837 (3)	0.063 (2)	0.750 (10)
H21A	0.476777	0.138625	0.577231	0.076*	0.750 (10)
C22A	0.5053 (9)	0.0610 (5)	0.6169 (3)	0.111 (4)	0.750 (10)
H22A	0.461813	0.033890	0.603836	0.166*	0.750 (10)
H22B	0.554510	0.035361	0.623918	0.166*	0.750 (10)
H22C	0.485590	0.080566	0.644212	0.166*	0.750 (10)
C23A	0.5941 (5)	0.1549 (4)	0.6028 (2)	0.077 (2)	0.750 (10)
H23A	0.607245	0.187683	0.580952	0.116*	0.750 (10)
H23B	0.574752	0.174977	0.630053	0.116*	0.750 (10)
H23C	0.643673	0.129773	0.609759	0.116*	0.750 (10)
C20B	0.547 (4)	0.0763 (14)	0.5354 (8)	0.054 (8)	0.250 (10)
H20C	0.584295	0.039966	0.530090	0.064*	0.250 (10)
H20D	0.489442	0.061991	0.529825	0.064*	0.250 (10)
C21B	0.5580 (16)	0.0986 (10)	0.5843 (8)	0.059 (5)	0.250 (10)
H21B	0.609574	0.124122	0.588993	0.071*	0.250 (10)
C22B	0.5585 (18)	0.0394 (12)	0.6138 (8)	0.083 (7)	0.250 (10)
H22D	0.607744	0.014005	0.607925	0.125*	0.250 (10)
H22E	0.559320	0.052021	0.645268	0.125*	0.250 (10)
H22F	0.508746	0.014214	0.607173	0.125*	0.250 (10)
C23B	0.481 (2)	0.1371 (14)	0.5941 (9)	0.109 (9)	0.250 (10)
H23D	0.479815	0.175203	0.575498	0.164*	0.250 (10)
H23E	0.431657	0.111512	0.587551	0.164*	0.250 (10)
H23F	0.482231	0.149319	0.625647	0.164*	0.250 (10)
C24A	0.6524 (10)	0.1416 (12)	0.4941 (9)	0.064 (5)	0.470 (14)
H24A	0.688292	0.127299	0.519406	0.077*	0.470 (14)
H24B	0.659136	0.188038	0.491366	0.077*	0.470 (14)
C25A	0.6827 (6)	0.1096 (5)	0.4504 (5)	0.059 (3)	0.470 (14)
H25A	0.654795	0.124917	0.422255	0.071*	0.470 (14)
C26A	0.7740 (10)	0.1322 (16)	0.4577 (12)	0.183 (12)	0.470 (14)
H26A	0.776972	0.178135	0.453310	0.275*	0.470 (14)
H26B	0.793109	0.121666	0.488048	0.275*	0.470 (14)
H26C	0.809276	0.110985	0.436207	0.275*	0.470 (14)
C27A	0.6801 (9)	0.0384 (5)	0.4573 (5)	0.078 (4)	0.470 (14)
H27A	0.698821	0.016938	0.430453	0.118*	0.470 (14)
H27B	0.716512	0.026877	0.482725	0.118*	0.470 (14)
H27C	0.623337	0.025307	0.463342	0.118*	0.470 (14)
C24B	0.6513 (7)	0.1498 (10)	0.4976 (7)	0.059 (4)	0.530 (14)
H24C	0.678451	0.159822	0.526744	0.070*	0.530 (14)
H24D	0.650592	0.188604	0.479149	0.070*	0.530 (14)
C25B	0.6999 (7)	0.0965 (7)	0.4739 (5)	0.105 (5)	0.530 (14)
H25B	0.717986	0.062556	0.495298	0.126*	0.530 (14)
C26B	0.7712 (16)	0.1200 (16)	0.4467 (9)	0.192 (10)	0.530 (14)
H26D	0.812795	0.140014	0.466502	0.288*	0.530 (14)
H26E	0.796299	0.084260	0.431236	0.288*	0.530 (14)
H26F	0.750760	0.151052	0.424746	0.288*	0.530 (14)
C27B	0.6489 (12)	0.0693 (10)	0.4344 (5)	0.161 (7)	0.530 (14)
H27D	0.680598	0.035889	0.419951	0.242*	0.530 (14)
H27E	0.597097	0.051561	0.445165	0.242*	0.530 (14)
H27F	0.636373	0.103079	0.412840	0.242*	0.530 (14)
C28	0.7268 (2)	0.18612 (19)	0.32102 (13)	0.0450 (8)	
C29	0.8473 (2)	0.1219 (2)	0.30255 (14)	0.0473 (9)	
H29A	0.864663	0.160720	0.286860	0.057*	
H29B	0.849911	0.086268	0.281175	0.057*	
C30	0.9082 (3)	0.1092 (2)	0.34129 (15)	0.0516 (9)	
H30	0.886331	0.073433	0.359361	0.062*	
C31	0.9196 (3)	0.1659 (2)	0.37135 (17)	0.0676 (13)	
H31A	0.945563	0.200296	0.354849	0.101*	
H31B	0.955037	0.154349	0.397101	0.101*	
H31C	0.865595	0.179910	0.381648	0.101*	
C32	0.9904 (3)	0.0886 (2)	0.32208 (17)	0.0589 (11)	
H32A	1.009490	0.121053	0.301377	0.088*	
H32B	0.982894	0.048415	0.306159	0.088*	
H32C	1.031575	0.083195	0.346352	0.088*	
C33	0.7123 (3)	0.07116 (19)	0.32141 (14)	0.0486 (9)	
H33A	0.663579	0.080740	0.339517	0.058*	
H33B	0.746555	0.039723	0.337995	0.058*	
C34	0.6825 (3)	0.0418 (2)	0.27712 (16)	0.0611 (11)	
H34	0.732168	0.026919	0.260969	0.073*	
C35	0.6364 (5)	0.0885 (3)	0.2475 (2)	0.095 (2)	
H35A	0.621329	0.068190	0.219048	0.142*	
H35B	0.671835	0.125195	0.242054	0.142*	
H35C	0.586147	0.102435	0.262172	0.142*	
C36	0.6301 (3)	−0.0155 (3)	0.2873 (2)	0.0721 (14)	
H36A	0.661772	−0.044595	0.306587	0.108*	
H36B	0.614341	−0.037055	0.259368	0.108*	
H36C	0.580121	−0.001939	0.302384	0.108*	
C37	0.5550 (2)	0.4200 (2)	0.15646 (13)	0.0463 (8)	
C38	0.6210 (3)	0.4313 (3)	0.08506 (14)	0.0626 (12)	
H38A	0.659391	0.461286	0.100354	0.075*	
H38B	0.603833	0.450337	0.056091	0.075*	
C39	0.6664 (4)	0.3695 (4)	0.0766 (2)	0.099 (2)	
H39	0.678260	0.348123	0.105786	0.118*	
C40	0.7466 (7)	0.3850 (6)	0.0555 (4)	0.196 (6)	
H40A	0.778858	0.346179	0.051997	0.294*	
H40B	0.777780	0.414839	0.074565	0.294*	
H40C	0.735669	0.404299	0.026243	0.294*	
C41	0.6212 (7)	0.3267 (5)	0.0479 (3)	0.165 (5)	
H41A	0.601261	0.349348	0.021175	0.247*	
H41B	0.574014	0.309483	0.063694	0.247*	
H41C	0.657569	0.291941	0.039120	0.247*	
C42	0.4660 (3)	0.4144 (3)	0.08963 (15)	0.0650 (12)	
H42A	0.436427	0.379367	0.104246	0.078*	
H42B	0.474902	0.401760	0.058341	0.078*	
C43	0.4119 (3)	0.4730 (3)	0.08980 (16)	0.0673 (13)	
H43	0.408728	0.488200	0.121277	0.081*	
C44	0.4477 (4)	0.5262 (3)	0.06206 (19)	0.0901 (19)	
H44A	0.448287	0.513341	0.030693	0.135*	
H44B	0.504371	0.535337	0.072662	0.135*	
H44C	0.413652	0.564319	0.064961	0.135*	
C45	0.3246 (4)	0.4543 (4)	0.0737 (2)	0.104 (2)	
H45A	0.326278	0.438921	0.042929	0.156*	
H45B	0.288067	0.491239	0.075007	0.156*	
H45C	0.303562	0.420712	0.092900	0.156*	
C46	0.6229 (2)	0.53121 (19)	0.40933 (13)	0.0462 (8)	
C47	0.7139 (3)	0.6055 (2)	0.44820 (15)	0.0560 (10)	
H47A	0.704518	0.651082	0.453985	0.067*	
H47B	0.745878	0.602027	0.420630	0.067*	
C48	0.7653 (3)	0.5773 (3)	0.48732 (19)	0.0777 (16)	
H48	0.739061	0.589709	0.515813	0.093*	
C49	0.7727 (5)	0.5081 (3)	0.4862 (2)	0.100 (2)	
H49A	0.717517	0.489013	0.487158	0.150*	
H49B	0.806092	0.493756	0.512068	0.150*	
H49C	0.799470	0.495186	0.458763	0.150*	
C50	0.8519 (4)	0.6084 (4)	0.4859 (2)	0.100 (2)	
H50A	0.885388	0.594678	0.511842	0.151*	
H50B	0.845993	0.654564	0.486388	0.151*	
H50C	0.879090	0.595578	0.458524	0.151*	
C51	0.5683 (3)	0.5879 (2)	0.47283 (14)	0.0552 (10)	
H51A	0.593420	0.608534	0.499573	0.066*	
H51B	0.543675	0.547415	0.482431	0.066*	
C52	0.5000 (3)	0.6301 (3)	0.45411 (17)	0.0654 (13)	
H52	0.476659	0.610009	0.426336	0.079*	
C53	0.5309 (4)	0.6957 (3)	0.44241 (19)	0.0772 (15)	
H53A	0.485136	0.720671	0.429452	0.116*	
H53B	0.574957	0.692130	0.420779	0.116*	
H53C	0.552500	0.716676	0.469436	0.116*	
C54	0.4316 (4)	0.6340 (3)	0.4879 (2)	0.093 (2)	
H54A	0.409528	0.591559	0.493263	0.140*	
H54B	0.387163	0.661505	0.476288	0.140*	
H54C	0.454065	0.651566	0.515970	0.140*	

### Atomic displacement parameters (Å^2^)

**Table:**

	*U*^11^	*U*^22^	*U*^33^	*U*^12^	*U*^13^	*U*^23^
Mo1	0.03888 (15)	0.04903 (17)	0.03986 (15)	−0.00657 (12)	0.00657 (12)	−0.00838 (12)
Mo2	0.03327 (13)	0.04424 (15)	0.03553 (14)	−0.00089 (11)	0.00322 (10)	−0.00169 (11)
Mo3	0.03619 (14)	0.03970 (15)	0.03931 (14)	0.00085 (11)	0.00285 (11)	−0.00202 (11)
Mo4	0.03519 (14)	0.04069 (15)	0.04030 (15)	−0.00056 (11)	0.00621 (11)	−0.00678 (11)
Mo5	0.03664 (14)	0.04272 (15)	0.03454 (14)	−0.00385 (11)	0.00392 (11)	−0.00701 (11)
Mo6	0.03491 (13)	0.04127 (15)	0.03495 (13)	−0.00051 (11)	0.00185 (10)	−0.00769 (11)
S1	0.0398 (4)	0.0570 (5)	0.0387 (4)	−0.0043 (4)	0.0029 (3)	0.0010 (4)
S2	0.0374 (4)	0.0667 (6)	0.0419 (5)	−0.0088 (4)	0.0004 (4)	−0.0028 (4)
S3	0.0353 (4)	0.0414 (4)	0.0517 (5)	−0.0003 (3)	0.0006 (4)	−0.0040 (4)
S4	0.0424 (4)	0.0472 (5)	0.0362 (4)	0.0020 (4)	0.0030 (3)	−0.0026 (4)
S5	0.0372 (4)	0.0507 (5)	0.0439 (5)	−0.0026 (4)	0.0070 (4)	−0.0061 (4)
S6	0.0466 (5)	0.0447 (5)	0.0604 (6)	0.0018 (4)	0.0092 (4)	−0.0095 (4)
S7	0.0424 (5)	0.0457 (5)	0.0429 (5)	−0.0041 (4)	0.0064 (4)	−0.0032 (4)
S8	0.0526 (5)	0.0577 (6)	0.0493 (5)	−0.0132 (4)	0.0130 (4)	−0.0166 (4)
S9	0.0603 (6)	0.0722 (7)	0.0458 (5)	−0.0215 (5)	0.0145 (5)	−0.0162 (5)
S10	0.0382 (4)	0.0559 (5)	0.0439 (5)	−0.0013 (4)	0.0071 (4)	0.0010 (4)
S11	0.0374 (4)	0.0496 (5)	0.0529 (5)	0.0025 (4)	0.0057 (4)	0.0028 (4)
S12	0.0481 (5)	0.0496 (5)	0.0596 (6)	0.0005 (4)	0.0019 (4)	0.0112 (4)
S13	0.0398 (4)	0.0477 (5)	0.0419 (4)	0.0018 (4)	−0.0001 (4)	0.0024 (4)
S14	0.0420 (4)	0.0456 (5)	0.0412 (4)	−0.0074 (4)	0.0052 (4)	−0.0092 (4)
S15	0.0492 (5)	0.0481 (5)	0.0434 (5)	−0.0051 (4)	0.0114 (4)	−0.0127 (4)
S16	0.0363 (4)	0.0479 (5)	0.0387 (4)	−0.0001 (4)	0.0028 (3)	−0.0046 (4)
S17	0.0440 (5)	0.0427 (5)	0.0424 (4)	−0.0028 (4)	0.0034 (4)	−0.0039 (4)
S18	0.0400 (4)	0.0456 (5)	0.0396 (4)	0.0003 (4)	0.0067 (3)	−0.0035 (4)
S19	0.0380 (4)	0.0529 (5)	0.0428 (5)	0.0020 (4)	−0.0016 (4)	−0.0052 (4)
S20	0.0351 (4)	0.0445 (5)	0.0429 (4)	−0.0030 (3)	0.0055 (3)	−0.0072 (4)
S21	0.0384 (5)	0.0453 (5)	0.0646 (6)	0.0016 (4)	0.0117 (4)	−0.0043 (4)
S22	0.0426 (5)	0.0448 (5)	0.0614 (6)	0.0013 (4)	0.0145 (4)	−0.0028 (4)
S23	0.0420 (5)	0.0541 (5)	0.0398 (4)	−0.0051 (4)	0.0064 (4)	−0.0050 (4)
S24	0.0424 (5)	0.0633 (6)	0.0388 (4)	−0.0077 (4)	0.0000 (4)	−0.0058 (4)
S25	0.0421 (5)	0.0509 (5)	0.0462 (5)	−0.0042 (4)	0.0022 (4)	−0.0154 (4)
S26	0.0429 (5)	0.0539 (5)	0.0431 (5)	0.0006 (4)	0.0048 (4)	−0.0142 (4)
S27	0.0404 (5)	0.0518 (5)	0.0467 (5)	−0.0040 (4)	0.0018 (4)	0.0028 (4)
N1	0.0468 (18)	0.075 (2)	0.0471 (19)	−0.0118 (17)	0.0069 (15)	−0.0211 (17)
N2	0.0390 (17)	0.062 (2)	0.0519 (19)	0.0063 (15)	0.0070 (14)	0.0028 (16)
N3	0.0492 (18)	0.0528 (19)	0.0412 (17)	0.0110 (15)	−0.0007 (14)	0.0027 (14)
N4	0.0397 (16)	0.0488 (18)	0.0498 (18)	0.0017 (14)	0.0066 (14)	−0.0078 (14)
N5	0.055 (2)	0.065 (2)	0.0346 (16)	−0.0013 (17)	0.0044 (14)	−0.0060 (15)
N6	0.0482 (18)	0.0518 (19)	0.0424 (17)	0.0009 (14)	−0.0017 (14)	−0.0149 (14)
C1	0.042 (2)	0.070 (3)	0.051 (2)	−0.0092 (19)	0.0108 (17)	−0.019 (2)
C2	0.047 (2)	0.060 (3)	0.051 (2)	−0.0028 (19)	−0.0005 (18)	−0.0159 (19)
C3	0.060 (3)	0.097 (4)	0.084 (4)	0.014 (3)	−0.012 (3)	−0.027 (3)
C4	0.070 (4)	0.139 (7)	0.112 (5)	0.028 (4)	0.001 (4)	−0.020 (5)
C5	0.098 (5)	0.088 (4)	0.077 (4)	0.026 (4)	−0.014 (3)	−0.002 (3)
C6	0.058 (3)	0.085 (3)	0.053 (2)	−0.017 (2)	0.016 (2)	−0.025 (2)
C7	0.071 (3)	0.081 (3)	0.051 (3)	−0.021 (3)	0.003 (2)	−0.010 (2)
C8	0.074 (3)	0.086 (4)	0.082 (4)	−0.009 (3)	−0.007 (3)	−0.007 (3)
C9	0.118 (5)	0.107 (5)	0.053 (3)	−0.014 (4)	0.007 (3)	−0.004 (3)
C10	0.0390 (19)	0.058 (2)	0.043 (2)	−0.0005 (17)	0.0041 (15)	−0.0041 (17)
C11A	0.048 (7)	0.083 (7)	0.058 (4)	0.005 (5)	0.009 (4)	0.008 (4)
C12A	0.052 (4)	0.117 (6)	0.064 (4)	0.014 (4)	0.012 (3)	0.003 (4)
C13A	0.077 (9)	0.207 (14)	0.064 (5)	−0.007 (8)	0.012 (6)	−0.010 (7)
C14A	0.048 (4)	0.175 (9)	0.070 (5)	0.005 (4)	0.013 (3)	0.027 (5)
C11B	0.024 (12)	0.093 (18)	0.067 (10)	−0.013 (12)	0.012 (10)	0.002 (10)
C12B	0.053 (9)	0.077 (10)	0.067 (9)	−0.006 (7)	0.018 (7)	−0.005 (8)
C13B	0.049 (14)	0.086 (11)	0.077 (14)	0.030 (11)	0.007 (12)	0.003 (11)
C14B	0.106 (17)	0.120 (15)	0.058 (11)	−0.045 (14)	0.022 (12)	0.003 (11)
C15	0.044 (2)	0.065 (3)	0.069 (3)	0.011 (2)	0.007 (2)	−0.001 (2)
C16	0.043 (2)	0.074 (3)	0.078 (3)	0.002 (2)	0.005 (2)	0.015 (3)
C17	0.065 (3)	0.083 (4)	0.069 (3)	−0.006 (3)	−0.007 (2)	0.013 (3)
C18	0.066 (3)	0.082 (4)	0.101 (4)	0.011 (3)	0.005 (3)	0.024 (3)
C19	0.052 (2)	0.045 (2)	0.0396 (19)	0.0079 (17)	0.0036 (16)	−0.0009 (15)
C20A	0.060 (6)	0.049 (5)	0.059 (5)	0.002 (4)	−0.002 (5)	0.015 (4)
C21A	0.071 (5)	0.070 (5)	0.050 (4)	0.020 (3)	0.008 (4)	0.020 (3)
C22A	0.157 (11)	0.092 (6)	0.084 (6)	0.024 (6)	0.030 (7)	0.037 (5)
C23A	0.095 (5)	0.085 (5)	0.052 (4)	0.025 (4)	−0.003 (3)	−0.008 (3)
C20B	0.055 (15)	0.060 (16)	0.045 (9)	0.021 (13)	−0.004 (9)	0.014 (9)
C21B	0.086 (13)	0.053 (10)	0.039 (8)	0.000 (9)	−0.012 (9)	0.025 (7)
C22B	0.097 (17)	0.084 (14)	0.067 (12)	−0.004 (11)	−0.022 (12)	0.051 (12)
C23B	0.17 (2)	0.094 (16)	0.069 (14)	0.053 (15)	0.025 (15)	0.008 (12)
C24A	0.075 (11)	0.060 (7)	0.057 (9)	0.008 (7)	−0.003 (8)	0.012 (7)
C25A	0.047 (5)	0.069 (6)	0.061 (6)	0.002 (4)	−0.015 (5)	0.012 (5)
C26A	0.057 (8)	0.155 (17)	0.34 (3)	−0.005 (10)	0.085 (12)	0.05 (2)
C27A	0.089 (9)	0.069 (6)	0.078 (8)	0.019 (6)	0.011 (7)	−0.010 (6)
C24B	0.031 (6)	0.101 (11)	0.044 (7)	0.004 (6)	0.003 (5)	0.016 (6)
C25B	0.084 (7)	0.156 (11)	0.078 (7)	0.072 (7)	0.032 (5)	0.041 (7)
C26B	0.181 (16)	0.23 (2)	0.166 (16)	0.060 (14)	0.138 (14)	−0.003 (15)
C27B	0.200 (14)	0.204 (18)	0.080 (9)	0.133 (12)	−0.016 (10)	−0.021 (10)
C28	0.0408 (19)	0.047 (2)	0.048 (2)	0.0020 (16)	0.0090 (16)	−0.0042 (16)
C29	0.0387 (19)	0.051 (2)	0.053 (2)	0.0071 (16)	0.0066 (16)	−0.0066 (17)
C30	0.046 (2)	0.051 (2)	0.057 (2)	0.0005 (17)	−0.0020 (18)	0.0014 (18)
C31	0.070 (3)	0.066 (3)	0.066 (3)	0.002 (2)	−0.011 (2)	−0.012 (2)
C32	0.043 (2)	0.061 (3)	0.073 (3)	−0.0007 (19)	−0.002 (2)	0.002 (2)
C33	0.047 (2)	0.042 (2)	0.058 (2)	0.0030 (16)	0.0100 (18)	−0.0040 (17)
C34	0.067 (3)	0.057 (3)	0.060 (3)	−0.003 (2)	0.002 (2)	−0.012 (2)
C35	0.140 (6)	0.068 (3)	0.075 (4)	−0.002 (4)	−0.036 (4)	−0.011 (3)
C36	0.063 (3)	0.070 (3)	0.084 (4)	−0.013 (2)	0.005 (3)	−0.014 (3)
C37	0.045 (2)	0.051 (2)	0.043 (2)	−0.0023 (17)	0.0014 (16)	−0.0066 (16)
C38	0.064 (3)	0.085 (3)	0.040 (2)	−0.002 (2)	0.0120 (19)	−0.006 (2)
C39	0.099 (5)	0.111 (5)	0.089 (4)	0.013 (4)	0.039 (4)	−0.026 (4)
C40	0.165 (10)	0.179 (11)	0.252 (14)	0.026 (8)	0.151 (10)	−0.015 (10)
C41	0.205 (12)	0.157 (9)	0.130 (8)	0.044 (8)	−0.009 (8)	−0.074 (7)
C42	0.069 (3)	0.085 (3)	0.040 (2)	−0.006 (3)	−0.006 (2)	−0.014 (2)
C43	0.057 (3)	0.099 (4)	0.046 (2)	0.008 (3)	−0.007 (2)	−0.010 (2)
C44	0.099 (4)	0.109 (5)	0.063 (3)	0.039 (4)	0.009 (3)	0.013 (3)
C45	0.073 (4)	0.146 (7)	0.092 (4)	0.012 (4)	−0.034 (3)	−0.035 (4)
C46	0.048 (2)	0.045 (2)	0.045 (2)	0.0072 (16)	−0.0049 (16)	−0.0102 (16)
C47	0.055 (2)	0.057 (2)	0.055 (2)	0.0014 (19)	−0.0022 (19)	−0.020 (2)
C48	0.060 (3)	0.096 (4)	0.076 (3)	0.014 (3)	−0.015 (3)	−0.033 (3)
C49	0.114 (5)	0.102 (5)	0.082 (4)	0.038 (4)	−0.032 (4)	−0.019 (4)
C50	0.059 (3)	0.147 (6)	0.095 (4)	0.009 (4)	−0.010 (3)	−0.049 (4)
C51	0.056 (2)	0.064 (3)	0.045 (2)	0.006 (2)	0.0027 (18)	−0.0198 (19)
C52	0.057 (3)	0.071 (3)	0.069 (3)	0.015 (2)	−0.002 (2)	−0.023 (2)
C53	0.078 (4)	0.083 (4)	0.071 (3)	0.021 (3)	0.001 (3)	−0.010 (3)
C54	0.064 (3)	0.093 (4)	0.124 (5)	0.008 (3)	0.025 (3)	−0.031 (4)

### Geometric parameters (Å, º)

**Table:**

Mo1—S7	2.3760 (10)	C13B—H13F	0.9800
Mo1—S5	2.3952 (9)	C14B—H14D	0.9800
Mo1—S1	2.3966 (11)	C14B—H14E	0.9800
Mo1—S8	2.4804 (11)	C14B—H14F	0.9800
Mo1—S6	2.4841 (11)	C15—C16	1.540 (7)
Mo1—S2	2.4889 (11)	C15—H15A	0.9900
Mo1—S9	2.5227 (10)	C15—H15B	0.9900
Mo1—Mo3	2.7194 (4)	C16—C17	1.507 (8)
Mo1—Mo2	2.7212 (4)	C16—C18	1.530 (7)
Mo2—S7	2.3815 (10)	C16—H16	1.0000
Mo2—S1	2.4039 (9)	C17—H17A	0.9800
Mo2—S3	2.4227 (9)	C17—H17B	0.9800
Mo2—S4	2.4595 (9)	C17—H17C	0.9800
Mo2—S10	2.4934 (9)	C18—H18A	0.9800
Mo2—S2	2.4963 (10)	C18—H18B	0.9800
Mo2—S11	2.5205 (10)	C18—H18C	0.9800
Mo2—Mo3	2.7020 (4)	C20A—C21A	1.541 (12)
Mo3—S7	2.3804 (10)	C20A—H20A	0.9900
Mo3—S5	2.4009 (9)	C20A—H20B	0.9900
Mo3—S3	2.4248 (10)	C21A—C23A	1.505 (10)
Mo3—S4	2.4595 (9)	C21A—C22A	1.522 (9)
Mo3—S12	2.4972 (11)	C21A—H21A	1.0000
Mo3—S6	2.4976 (10)	C22A—H22A	0.9800
Mo3—S13	2.5224 (10)	C22A—H22B	0.9800
Mo4—S20	2.3832 (9)	C22A—H22C	0.9800
Mo4—S18	2.4030 (9)	C23A—H23A	0.9800
Mo4—S14	2.4040 (10)	C23A—H23B	0.9800
Mo4—S19	2.4754 (10)	C23A—H23C	0.9800
Mo4—S15	2.4815 (10)	C20B—C21B	1.549 (16)
Mo4—S21	2.4877 (10)	C20B—H20C	0.9900
Mo4—S22	2.5074 (11)	C20B—H20D	0.9900
Mo4—Mo6	2.7077 (4)	C21B—C23B	1.521 (15)
Mo4—Mo5	2.7086 (4)	C21B—C22B	1.531 (14)
Mo5—S20	2.3845 (9)	C21B—H21B	1.0000
Mo5—S16	2.4021 (9)	C22B—H22D	0.9800
Mo5—S14	2.4027 (10)	C22B—H22E	0.9800
Mo5—S15	2.4760 (10)	C22B—H22F	0.9800
Mo5—S17	2.4889 (10)	C23B—H23D	0.9800
Mo5—S23	2.4896 (10)	C23B—H23E	0.9800
Mo5—S24	2.5174 (10)	C23B—H23F	0.9800
Mo5—Mo6	2.7136 (4)	C24A—C25A	1.566 (15)
Mo6—S20	2.3870 (9)	C24A—H24A	0.9900
Mo6—S16	2.3947 (9)	C24A—H24B	0.9900
Mo6—S18	2.3978 (10)	C25A—C27A	1.519 (12)
Mo6—S17	2.4744 (10)	C25A—C26A	1.561 (13)
Mo6—S25	2.4802 (10)	C25A—H25A	1.0000
Mo6—S19	2.4805 (10)	C26A—H26A	0.9800
Mo6—S26	2.5193 (9)	C26A—H26B	0.9800
S1—S2	2.0671 (13)	C26A—H26C	0.9800
S1—S27	2.7810 (13)	C27A—H27A	0.9800
S3—S4	2.2414 (13)	C27A—H27B	0.9800
S3—S27	2.4849 (14)	C27A—H27C	0.9800
S5—S6	2.0725 (15)	C24B—C25B	1.555 (14)
S5—S27	2.8077 (14)	C24B—H24C	0.9900
S8—C1	1.722 (4)	C24B—H24D	0.9900
S9—C1	1.728 (5)	C25B—C27B	1.538 (13)
S10—C10	1.710 (4)	C25B—C26B	1.515 (13)
S11—C10	1.724 (4)	C25B—H25B	1.0000
S12—C19	1.718 (4)	C26B—H26D	0.9800
S13—C19	1.713 (4)	C26B—H26E	0.9800
S14—S15	2.0878 (13)	C26B—H26F	0.9800
S14—S27	2.7767 (13)	C27B—H27D	0.9800
S16—S17	2.0813 (13)	C27B—H27E	0.9800
S16—S27	2.7494 (13)	C27B—H27F	0.9800
S18—S19	2.1198 (13)	C29—C30	1.527 (6)
S18—S27	2.7252 (13)	C29—H29A	0.9900
S21—C28	1.725 (4)	C29—H29B	0.9900
S22—C28	1.718 (4)	C30—C31	1.507 (6)
S23—C37	1.720 (4)	C30—C32	1.526 (6)
S24—C37	1.713 (4)	C30—H30	1.0000
S25—C46	1.734 (4)	C31—H31A	0.9800
S26—C46	1.711 (4)	C31—H31B	0.9800
N1—C1	1.318 (5)	C31—H31C	0.9800
N1—C2	1.469 (6)	C32—H32A	0.9800
N1—C6	1.482 (6)	C32—H32B	0.9800
N2—C10	1.336 (5)	C32—H32C	0.9800
N2—C15	1.465 (6)	C33—C34	1.533 (6)
N2—C11A	1.474 (8)	C33—H33A	0.9900
N2—C11B	1.479 (14)	C33—H33B	0.9900
N3—C19	1.331 (5)	C34—C35	1.511 (8)
N3—C24A	1.463 (12)	C34—C36	1.512 (7)
N3—C20B	1.472 (15)	C34—H34	1.0000
N3—C24B	1.468 (10)	C35—H35A	0.9800
N3—C20A	1.472 (7)	C35—H35B	0.9800
N4—C28	1.323 (5)	C35—H35C	0.9800
N4—C29	1.474 (5)	C36—H36A	0.9800
N4—C33	1.476 (5)	C36—H36B	0.9800
N5—C37	1.326 (5)	C36—H36C	0.9800
N5—C42	1.475 (6)	C38—C39	1.522 (8)
N5—C38	1.476 (5)	C38—H38A	0.9900
N6—C46	1.327 (5)	C38—H38B	0.9900
N6—C47	1.460 (5)	C39—C41	1.436 (11)
N6—C51	1.474 (5)	C39—C40	1.493 (10)
C2—C3	1.527 (7)	C39—H39	1.0000
C2—H2A	0.9900	C40—H40A	0.9800
C2—H2B	0.9900	C40—H40B	0.9800
C3—C4	1.503 (8)	C40—H40C	0.9800
C3—C5	1.509 (9)	C41—H41A	0.9800
C3—H3	1.0000	C41—H41B	0.9800
C4—H4A	0.9800	C41—H41C	0.9800
C4—H4B	0.9800	C42—C43	1.516 (7)
C4—H4C	0.9800	C42—H42A	0.9900
C5—H5A	0.9800	C42—H42B	0.9900
C5—H5B	0.9800	C43—C44	1.523 (8)
C5—H5C	0.9800	C43—C45	1.531 (7)
C6—C7	1.539 (7)	C43—H43	1.0000
C6—H6A	0.9900	C44—H44A	0.9800
C6—H6B	0.9900	C44—H44B	0.9800
C7—C8	1.522 (8)	C44—H44C	0.9800
C7—C9	1.532 (7)	C45—H45A	0.9800
C7—H7	1.0000	C45—H45B	0.9800
C8—H8A	0.9800	C45—H45C	0.9800
C8—H8B	0.9800	C47—C48	1.539 (7)
C8—H8C	0.9800	C47—H47A	0.9900
C9—H9A	0.9800	C47—H47B	0.9900
C9—H9B	0.9800	C48—C49	1.466 (9)
C9—H9C	0.9800	C48—C50	1.548 (8)
C11A—C12A	1.529 (13)	C48—H48	1.0000
C11A—H11A	0.9900	C49—H49A	0.9800
C11A—H11B	0.9900	C49—H49B	0.9800
C12A—C14A	1.525 (9)	C49—H49C	0.9800
C12A—C13A	1.525 (12)	C50—H50A	0.9800
C12A—H12A	1.0000	C50—H50B	0.9800
C13A—H13A	0.9800	C50—H50C	0.9800
C13A—H13B	0.9800	C51—C52	1.515 (7)
C13A—H13C	0.9800	C51—H51A	0.9900
C14A—H14A	0.9800	C51—H51B	0.9900
C14A—H14B	0.9800	C52—C53	1.516 (8)
C14A—H14C	0.9800	C52—C54	1.524 (7)
C11B—C12B	1.544 (17)	C52—H52	1.0000
C11B—H11C	0.9900	C53—H53A	0.9800
C11B—H11D	0.9900	C53—H53B	0.9800
C12B—C14B	1.519 (14)	C53—H53C	0.9800
C12B—C13B	1.521 (15)	C54—H54A	0.9800
C12B—H12B	1.0000	C54—H54B	0.9800
C13B—H13D	0.9800	C54—H54C	0.9800
C13B—H13E	0.9800		
			
S7—Mo1—S5	110.37 (3)	H5B—C5—H5C	109.5
S7—Mo1—S1	110.21 (3)	N1—C6—C7	112.6 (4)
S5—Mo1—S1	83.01 (3)	N1—C6—H6A	109.1
S7—Mo1—S8	85.33 (3)	C7—C6—H6A	109.1
S5—Mo1—S8	132.53 (4)	N1—C6—H6B	109.1
S1—Mo1—S8	134.71 (4)	C7—C6—H6B	109.1
S7—Mo1—S6	86.30 (4)	H6A—C6—H6B	107.8
S5—Mo1—S6	50.23 (3)	C8—C7—C9	111.4 (5)
S1—Mo1—S6	133.05 (3)	C8—C7—C6	112.0 (4)
S8—Mo1—S6	88.64 (4)	C9—C7—C6	108.7 (5)
S7—Mo1—S2	85.24 (3)	C8—C7—H7	108.2
S5—Mo1—S2	132.71 (4)	C9—C7—H7	108.2
S1—Mo1—S2	50.02 (3)	C6—C7—H7	108.2
S8—Mo1—S2	91.69 (4)	C7—C8—H8A	109.5
S6—Mo1—S2	171.47 (4)	C7—C8—H8B	109.5
S7—Mo1—S9	155.30 (4)	H8A—C8—H8B	109.5
S5—Mo1—S9	87.45 (3)	C7—C8—H8C	109.5
S1—Mo1—S9	88.23 (4)	H8A—C8—H8C	109.5
S8—Mo1—S9	69.97 (4)	H8B—C8—H8C	109.5
S6—Mo1—S9	92.94 (4)	C7—C9—H9A	109.5
S2—Mo1—S9	95.18 (4)	C7—C9—H9B	109.5
S7—Mo1—Mo3	55.20 (2)	H9A—C9—H9B	109.5
S5—Mo1—Mo3	55.56 (2)	C7—C9—H9C	109.5
S1—Mo1—Mo3	95.87 (3)	H9A—C9—H9C	109.5
S8—Mo1—Mo3	126.03 (3)	H9B—C9—H9C	109.5
S6—Mo1—Mo3	57.15 (2)	N2—C10—S10	123.8 (3)
S2—Mo1—Mo3	116.48 (3)	N2—C10—S11	123.4 (3)
S9—Mo1—Mo3	141.69 (3)	S10—C10—S11	112.8 (2)
S7—Mo1—Mo2	55.21 (2)	N2—C11A—C12A	113.0 (9)
S5—Mo1—Mo2	94.78 (2)	N2—C11A—H11A	109.0
S1—Mo1—Mo2	55.59 (2)	C12A—C11A—H11A	109.0
S8—Mo1—Mo2	128.45 (3)	N2—C11A—H11B	109.0
S6—Mo1—Mo2	116.66 (3)	C12A—C11A—H11B	109.0
S2—Mo1—Mo2	57.05 (2)	H11A—C11A—H11B	107.8
S9—Mo1—Mo2	142.98 (4)	C11A—C12A—C14A	107.7 (7)
Mo3—Mo1—Mo2	59.555 (11)	C11A—C12A—C13A	111.6 (9)
S7—Mo2—S1	109.77 (3)	C14A—C12A—C13A	111.1 (9)
S7—Mo2—S3	111.29 (3)	C11A—C12A—H12A	108.8
S1—Mo2—S3	82.57 (3)	C14A—C12A—H12A	108.8
S7—Mo2—S4	83.35 (3)	C13A—C12A—H12A	108.8
S1—Mo2—S4	136.71 (3)	C12A—C13A—H13A	109.5
S3—Mo2—S4	54.65 (3)	C12A—C13A—H13B	109.5
S7—Mo2—S10	87.16 (3)	H13A—C13A—H13B	109.5
S1—Mo2—S10	130.54 (3)	C12A—C13A—H13C	109.5
S3—Mo2—S10	135.26 (3)	H13A—C13A—H13C	109.5
S4—Mo2—S10	89.85 (3)	H13B—C13A—H13C	109.5
S7—Mo2—S2	84.96 (4)	C12A—C14A—H14A	109.5
S1—Mo2—S2	49.86 (3)	C12A—C14A—H14B	109.5
S3—Mo2—S2	132.19 (3)	H14A—C14A—H14B	109.5
S4—Mo2—S2	168.22 (4)	C12A—C14A—H14C	109.5
S10—Mo2—S2	88.12 (3)	H14A—C14A—H14C	109.5
S7—Mo2—S11	156.66 (3)	H14B—C14A—H14C	109.5
S1—Mo2—S11	85.60 (3)	N2—C11B—C12B	120.9 (18)
S3—Mo2—S11	87.46 (3)	N2—C11B—H11C	107.1
S4—Mo2—S11	97.57 (3)	C12B—C11B—H11C	107.1
S10—Mo2—S11	69.55 (3)	N2—C11B—H11D	107.1
S2—Mo2—S11	92.60 (4)	C12B—C11B—H11D	107.1
S7—Mo2—Mo3	55.41 (2)	H11C—C11B—H11D	106.8
S1—Mo2—Mo3	96.16 (3)	C14B—C12B—C13B	111 (2)
S3—Mo2—Mo3	56.16 (2)	C14B—C12B—C11B	113.0 (16)
S4—Mo2—Mo3	56.68 (2)	C13B—C12B—C11B	109 (3)
S10—Mo2—Mo3	129.53 (3)	C14B—C12B—H12B	107.9
S2—Mo2—Mo3	116.85 (3)	C13B—C12B—H12B	107.9
S11—Mo2—Mo3	142.76 (3)	C11B—C12B—H12B	107.9
S7—Mo2—Mo1	55.02 (2)	C12B—C13B—H13D	109.5
S1—Mo2—Mo1	55.34 (3)	C12B—C13B—H13E	109.5
S3—Mo2—Mo1	95.18 (2)	H13D—C13B—H13E	109.5
S4—Mo2—Mo1	116.47 (2)	C12B—C13B—H13F	109.5
S10—Mo2—Mo1	127.29 (3)	H13D—C13B—H13F	109.5
S2—Mo2—Mo1	56.79 (3)	H13E—C13B—H13F	109.5
S11—Mo2—Mo1	139.90 (3)	C12B—C14B—H14D	109.5
Mo3—Mo2—Mo1	60.190 (11)	C12B—C14B—H14E	109.5
S7—Mo3—S5	110.02 (3)	H14D—C14B—H14E	109.5
S7—Mo3—S3	111.26 (3)	C12B—C14B—H14F	109.5
S5—Mo3—S3	81.18 (3)	H14D—C14B—H14F	109.5
S7—Mo3—S4	83.37 (3)	H14E—C14B—H14F	109.5
S5—Mo3—S4	135.29 (3)	N2—C15—C16	114.2 (4)
S3—Mo3—S4	54.63 (3)	N2—C15—H15A	108.7
S7—Mo3—S12	85.92 (4)	C16—C15—H15A	108.7
S5—Mo3—S12	132.51 (4)	N2—C15—H15B	108.7
S3—Mo3—S12	135.67 (4)	C16—C15—H15B	108.7
S4—Mo3—S12	89.52 (4)	H15A—C15—H15B	107.6
S7—Mo3—S6	85.90 (3)	C17—C16—C18	111.2 (5)
S5—Mo3—S6	50.01 (3)	C17—C16—C15	112.7 (4)
S3—Mo3—S6	131.03 (4)	C18—C16—C15	107.6 (5)
S4—Mo3—S6	169.25 (3)	C17—C16—H16	108.4
S12—Mo3—S6	89.12 (4)	C18—C16—H16	108.4
S7—Mo3—S13	155.49 (3)	C15—C16—H16	108.4
S5—Mo3—S13	86.07 (3)	C16—C17—H17A	109.5
S3—Mo3—S13	88.87 (3)	C16—C17—H17B	109.5
S4—Mo3—S13	98.30 (3)	H17A—C17—H17B	109.5
S12—Mo3—S13	69.69 (3)	C16—C17—H17C	109.5
S6—Mo3—S13	91.22 (3)	H17A—C17—H17C	109.5
S7—Mo3—Mo2	55.45 (3)	H17B—C17—H17C	109.5
S5—Mo3—Mo2	95.14 (3)	C16—C18—H18A	109.5
S3—Mo3—Mo2	56.09 (2)	C16—C18—H18B	109.5
S4—Mo3—Mo2	56.68 (2)	H18A—C18—H18B	109.5
S12—Mo3—Mo2	128.46 (3)	C16—C18—H18C	109.5
S6—Mo3—Mo2	116.88 (3)	H18A—C18—H18C	109.5
S13—Mo3—Mo2	144.05 (3)	H18B—C18—H18C	109.5
S7—Mo3—Mo1	55.05 (2)	N3—C19—S13	123.3 (3)
S5—Mo3—Mo1	55.36 (2)	N3—C19—S12	123.3 (3)
S3—Mo3—Mo1	95.18 (3)	S13—C19—S12	113.4 (2)
S4—Mo3—Mo1	116.54 (2)	N3—C20A—C21A	112.2 (9)
S12—Mo3—Mo1	126.46 (3)	N3—C20A—H20A	109.2
S6—Mo3—Mo1	56.68 (3)	C21A—C20A—H20A	109.2
S13—Mo3—Mo1	139.79 (2)	N3—C20A—H20B	109.2
Mo2—Mo3—Mo1	60.255 (11)	C21A—C20A—H20B	109.2
S20—Mo4—S18	110.65 (3)	H20A—C20A—H20B	107.9
S20—Mo4—S14	110.68 (3)	C23A—C21A—C22A	110.7 (8)
S18—Mo4—S14	81.96 (3)	C23A—C21A—C20A	111.3 (10)
S20—Mo4—S19	85.22 (3)	C22A—C21A—C20A	109.1 (7)
S18—Mo4—S19	51.48 (3)	C23A—C21A—H21A	108.5
S14—Mo4—S19	133.18 (3)	C22A—C21A—H21A	108.5
S20—Mo4—S15	85.82 (3)	C20A—C21A—H21A	108.5
S18—Mo4—S15	132.29 (3)	C21A—C22A—H22A	109.5
S14—Mo4—S15	50.57 (3)	C21A—C22A—H22B	109.5
S19—Mo4—S15	171.03 (4)	H22A—C22A—H22B	109.5
S20—Mo4—S21	85.76 (3)	C21A—C22A—H22C	109.5
S18—Mo4—S21	136.19 (4)	H22A—C22A—H22C	109.5
S14—Mo4—S21	131.32 (3)	H22B—C22A—H22C	109.5
S19—Mo4—S21	92.19 (3)	C21A—C23A—H23A	109.5
S15—Mo4—S21	87.61 (4)	C21A—C23A—H23B	109.5
S20—Mo4—S22	155.79 (3)	H23A—C23A—H23B	109.5
S18—Mo4—S22	87.64 (3)	C21A—C23A—H23C	109.5
S14—Mo4—S22	86.82 (4)	H23A—C23A—H23C	109.5
S19—Mo4—S22	94.91 (4)	H23B—C23A—H23C	109.5
S15—Mo4—S22	93.45 (4)	N3—C20B—C21B	110.8 (18)
S21—Mo4—S22	70.04 (3)	N3—C20B—H20C	109.5
S20—Mo4—Mo6	55.48 (2)	C21B—C20B—H20C	109.5
S18—Mo4—Mo6	55.58 (2)	N3—C20B—H20D	109.5
S14—Mo4—Mo6	95.29 (3)	C21B—C20B—H20D	109.5
S19—Mo4—Mo6	56.97 (2)	H20C—C20B—H20D	108.1
S15—Mo4—Mo6	116.84 (3)	C23B—C21B—C22B	108 (2)
S21—Mo4—Mo6	129.28 (3)	C23B—C21B—C20B	106 (3)
S22—Mo4—Mo6	142.21 (3)	C22B—C21B—C20B	107.4 (18)
S20—Mo4—Mo5	55.40 (2)	C23B—C21B—H21B	111.6
S18—Mo4—Mo5	95.33 (3)	C22B—C21B—H21B	111.6
S14—Mo4—Mo5	55.68 (2)	C20B—C21B—H21B	111.6
S19—Mo4—Mo5	116.96 (3)	C21B—C22B—H22D	109.5
S15—Mo4—Mo5	56.78 (3)	C21B—C22B—H22E	109.5
S21—Mo4—Mo5	125.82 (3)	H22D—C22B—H22E	109.5
S22—Mo4—Mo5	141.29 (3)	C21B—C22B—H22F	109.5
Mo6—Mo4—Mo5	60.132 (11)	H22D—C22B—H22F	109.5
S20—Mo5—S16	110.16 (3)	H22E—C22B—H22F	109.5
S20—Mo5—S14	110.68 (3)	C21B—C23B—H23D	109.5
S16—Mo5—S14	83.54 (3)	C21B—C23B—H23E	109.5
S20—Mo5—S15	85.92 (3)	H23D—C23B—H23E	109.5
S16—Mo5—S15	133.96 (3)	C21B—C23B—H23F	109.5
S14—Mo5—S15	50.65 (3)	H23D—C23B—H23F	109.5
S20—Mo5—S17	84.36 (3)	H23E—C23B—H23F	109.5
S16—Mo5—S17	50.33 (3)	N3—C24A—C25A	114.4 (12)
S14—Mo5—S17	133.49 (3)	N3—C24A—H24A	108.7
S15—Mo5—S17	170.28 (3)	C25A—C24A—H24A	108.7
S20—Mo5—S23	86.52 (3)	N3—C24A—H24B	108.7
S16—Mo5—S23	134.78 (4)	C25A—C24A—H24B	108.7
S14—Mo5—S23	130.77 (3)	H24A—C24A—H24B	107.6
S15—Mo5—S23	87.26 (3)	C27A—C25A—C24A	107.6 (14)
S17—Mo5—S23	92.50 (3)	C27A—C25A—C26A	108.3 (16)
S20—Mo5—S24	156.51 (3)	C24A—C25A—C26A	93.9 (14)
S16—Mo5—S24	87.44 (3)	C27A—C25A—H25A	115.0
S14—Mo5—S24	85.97 (3)	C24A—C25A—H25A	115.0
S15—Mo5—S24	92.88 (4)	C26A—C25A—H25A	115.0
S17—Mo5—S24	96.18 (3)	C25A—C26A—H26A	109.5
S23—Mo5—S24	69.99 (3)	C25A—C26A—H26B	109.4
S20—Mo5—Mo4	55.36 (2)	H26A—C26A—H26B	109.5
S16—Mo5—Mo4	96.30 (2)	C25A—C26A—H26C	109.5
S14—Mo5—Mo4	55.73 (2)	H26A—C26A—H26C	109.5
S15—Mo5—Mo4	56.98 (3)	H26B—C26A—H26C	109.5
S17—Mo5—Mo4	116.32 (2)	C25A—C27A—H27A	109.5
S23—Mo5—Mo4	126.20 (3)	C25A—C27A—H27B	109.5
S24—Mo5—Mo4	140.55 (3)	H27A—C27A—H27B	109.5
S20—Mo5—Mo6	55.38 (2)	C25A—C27A—H27C	109.5
S16—Mo5—Mo6	55.42 (2)	H27A—C27A—H27C	109.5
S14—Mo5—Mo6	95.17 (2)	H27B—C27A—H27C	109.5
S15—Mo5—Mo6	116.82 (3)	N3—C24B—C25B	108.9 (11)
S17—Mo5—Mo6	56.60 (2)	N3—C24B—H24C	109.9
S23—Mo5—Mo6	130.10 (3)	C25B—C24B—H24C	109.9
S24—Mo5—Mo6	142.27 (3)	N3—C24B—H24D	109.9
Mo4—Mo5—Mo6	59.918 (11)	C25B—C24B—H24D	109.9
S20—Mo6—S16	110.33 (3)	H24C—C24B—H24D	108.3
S20—Mo6—S18	110.70 (3)	C27B—C25B—C24B	110.8 (12)
S16—Mo6—S18	83.64 (3)	C27B—C25B—C26B	96.1 (17)
S20—Mo6—S17	84.63 (3)	C24B—C25B—C26B	114.3 (15)
S16—Mo6—S17	50.58 (3)	C27B—C25B—H25B	111.6
S18—Mo6—S17	133.89 (3)	C24B—C25B—H25B	111.6
S20—Mo6—S25	87.21 (3)	C26B—C25B—H25B	111.5
S16—Mo6—S25	131.46 (4)	C25B—C26B—H26D	109.5
S18—Mo6—S25	133.28 (3)	C25B—C26B—H26E	109.5
S17—Mo6—S25	88.92 (3)	H26D—C26B—H26E	109.5
S20—Mo6—S19	85.03 (3)	C25B—C26B—H26F	109.4
S16—Mo6—S19	134.84 (3)	H26D—C26B—H26F	109.5
S18—Mo6—S19	51.48 (3)	H26E—C26B—H26F	109.5
S17—Mo6—S19	169.64 (3)	C25B—C27B—H27D	109.5
S25—Mo6—S19	89.95 (3)	C25B—C27B—H27E	109.5
S20—Mo6—S26	157.33 (3)	H27D—C27B—H27E	109.5
S16—Mo6—S26	86.35 (3)	C25B—C27B—H27F	109.5
S18—Mo6—S26	85.64 (3)	H27D—C27B—H27F	109.5
S17—Mo6—S26	95.31 (3)	H27E—C27B—H27F	109.5
S25—Mo6—S26	70.13 (3)	N4—C28—S22	123.1 (3)
S19—Mo6—S26	93.99 (3)	N4—C28—S21	124.1 (3)
S20—Mo6—Mo4	55.35 (2)	S22—C28—S21	112.8 (2)
S16—Mo6—Mo4	96.50 (2)	N4—C29—C30	113.9 (3)
S18—Mo6—Mo4	55.76 (2)	N4—C29—H29A	108.8
S17—Mo6—Mo4	116.87 (2)	C30—C29—H29A	108.8
S25—Mo6—Mo4	128.78 (3)	N4—C29—H29B	108.8
S19—Mo6—Mo4	56.79 (2)	C30—C29—H29B	108.8
S26—Mo6—Mo4	140.45 (3)	H29A—C29—H29B	107.7
S20—Mo6—Mo5	55.30 (2)	C31—C30—C32	111.0 (4)
S16—Mo6—Mo5	55.68 (2)	C31—C30—C29	112.5 (4)
S18—Mo6—Mo5	95.32 (2)	C32—C30—C29	108.2 (4)
S17—Mo6—Mo5	57.12 (2)	C31—C30—H30	108.3
S25—Mo6—Mo5	128.55 (3)	C32—C30—H30	108.3
S19—Mo6—Mo5	116.60 (2)	C29—C30—H30	108.3
S26—Mo6—Mo5	141.47 (3)	C30—C31—H31A	109.5
Mo4—Mo6—Mo5	59.950 (11)	C30—C31—H31B	109.5
S2—S1—Mo1	67.31 (4)	H31A—C31—H31B	109.5
S2—S1—Mo2	67.40 (4)	C30—C31—H31C	109.5
Mo1—S1—Mo2	69.06 (3)	H31A—C31—H31C	109.5
S2—S1—S27	164.31 (6)	H31B—C31—H31C	109.5
Mo1—S1—S27	103.80 (4)	C30—C32—H32A	109.5
Mo2—S1—S27	97.62 (4)	C30—C32—H32B	109.5
S1—S2—Mo1	62.67 (4)	H32A—C32—H32B	109.5
S1—S2—Mo2	62.75 (4)	C30—C32—H32C	109.5
Mo1—S2—Mo2	66.17 (3)	H32A—C32—H32C	109.5
S4—S3—Mo2	63.51 (3)	H32B—C32—H32C	109.5
S4—S3—Mo3	63.47 (3)	N4—C33—C34	113.7 (4)
Mo2—S3—Mo3	67.75 (3)	N4—C33—H33A	108.8
S4—S3—S27	167.49 (5)	C34—C33—H33A	108.8
Mo2—S3—S27	105.62 (4)	N4—C33—H33B	108.8
Mo3—S3—S27	107.55 (4)	C34—C33—H33B	108.8
S3—S4—Mo2	61.84 (3)	H33A—C33—H33B	107.7
S3—S4—Mo3	61.90 (3)	C35—C34—C36	111.7 (5)
Mo2—S4—Mo3	66.64 (2)	C35—C34—C33	112.6 (4)
S6—S5—Mo1	67.11 (4)	C36—C34—C33	108.3 (4)
S6—S5—Mo3	67.42 (4)	C35—C34—H34	108.0
Mo1—S5—Mo3	69.08 (3)	C36—C34—H34	108.0
S6—S5—S27	164.88 (5)	C33—C34—H34	108.0
Mo1—S5—S27	103.05 (4)	C34—C35—H35A	109.5
Mo3—S5—S27	98.71 (4)	C34—C35—H35B	109.5
S5—S6—Mo1	62.66 (4)	H35A—C35—H35B	109.5
S5—S6—Mo3	62.57 (4)	C34—C35—H35C	109.5
Mo1—S6—Mo3	66.17 (3)	H35A—C35—H35C	109.5
Mo1—S7—Mo3	69.74 (3)	H35B—C35—H35C	109.5
Mo1—S7—Mo2	69.78 (3)	C34—C36—H36A	109.5
Mo3—S7—Mo2	69.14 (3)	C34—C36—H36B	109.5
C1—S8—Mo1	89.31 (15)	H36A—C36—H36B	109.5
C1—S9—Mo1	87.81 (14)	C34—C36—H36C	109.5
C10—S10—Mo2	89.00 (13)	H36A—C36—H36C	109.5
C10—S11—Mo2	87.80 (15)	H36B—C36—H36C	109.5
C19—S12—Mo3	88.61 (14)	N5—C37—S24	122.9 (3)
C19—S13—Mo3	87.90 (15)	N5—C37—S23	123.6 (3)
S15—S14—Mo5	66.50 (4)	S24—C37—S23	113.5 (2)
S15—S14—Mo4	66.64 (4)	N5—C38—C39	113.0 (5)
Mo5—S14—Mo4	68.60 (3)	N5—C38—H38A	109.0
S15—S14—S27	166.72 (5)	C39—C38—H38A	109.0
Mo5—S14—S27	102.37 (4)	N5—C38—H38B	109.0
Mo4—S14—S27	103.17 (4)	C39—C38—H38B	109.0
S14—S15—Mo5	62.86 (4)	H38A—C38—H38B	107.8
S14—S15—Mo4	62.79 (4)	C41—C39—C40	108.5 (8)
Mo5—S15—Mo4	66.24 (2)	C41—C39—C38	113.6 (7)
S17—S16—Mo6	66.69 (4)	C40—C39—C38	108.1 (7)
S17—S16—Mo5	67.00 (4)	C41—C39—H39	108.8
Mo6—S16—Mo5	68.90 (3)	C40—C39—H39	108.8
S17—S16—S27	166.89 (5)	C38—C39—H39	108.8
Mo6—S16—S27	102.14 (4)	C39—C40—H40A	109.5
Mo5—S16—S27	103.19 (4)	C39—C40—H40B	109.5
S16—S17—Mo6	62.73 (4)	H40A—C40—H40B	109.5
S16—S17—Mo5	62.67 (3)	C39—C40—H40C	109.5
Mo6—S17—Mo5	66.28 (3)	H40A—C40—H40C	109.5
S19—S18—Mo6	66.27 (4)	H40B—C40—H40C	109.5
S19—S18—Mo4	66.02 (4)	C39—C41—H41A	109.5
Mo6—S18—Mo4	68.67 (3)	C39—C41—H41B	109.5
S19—S18—S27	167.32 (5)	H41A—C41—H41B	109.5
Mo6—S18—S27	102.76 (4)	C39—C41—H41C	109.5
Mo4—S18—S27	104.73 (4)	H41A—C41—H41C	109.5
S18—S19—Mo4	62.49 (3)	H41B—C41—H41C	109.5
S18—S19—Mo6	62.25 (4)	N5—C42—C43	113.9 (4)
Mo4—S19—Mo6	66.24 (3)	N5—C42—H42A	108.8
Mo4—S20—Mo5	69.24 (3)	C43—C42—H42A	108.8
Mo4—S20—Mo6	69.17 (3)	N5—C42—H42B	108.8
Mo5—S20—Mo6	69.32 (3)	C43—C42—H42B	108.8
C28—S21—Mo4	88.05 (13)	H42A—C42—H42B	107.7
C28—S22—Mo4	87.56 (14)	C42—C43—C44	111.8 (4)
C37—S23—Mo5	88.31 (14)	C42—C43—C45	108.4 (5)
C37—S24—Mo5	87.56 (14)	C44—C43—C45	112.3 (5)
C46—S25—Mo6	88.83 (14)	C42—C43—H43	108.0
C46—S26—Mo6	88.05 (13)	C44—C43—H43	108.0
S3—S27—S18	81.65 (4)	C45—C43—H43	108.0
S3—S27—S16	128.52 (5)	C43—C44—H44A	109.5
S18—S27—S16	71.43 (4)	C43—C44—H44B	109.5
S3—S27—S14	138.13 (5)	H44A—C44—H44B	109.5
S18—S27—S14	69.91 (3)	C43—C44—H44C	109.5
S16—S27—S14	70.79 (3)	H44A—C44—H44C	109.5
S3—S27—S1	74.18 (4)	H44B—C44—H44C	109.5
S18—S27—S1	145.90 (5)	C43—C45—H45A	109.5
S16—S27—S1	142.67 (5)	C43—C45—H45B	109.5
S14—S27—S1	114.24 (4)	H45A—C45—H45B	109.5
S3—S27—S5	72.49 (4)	C43—C45—H45C	109.5
S18—S27—S5	80.85 (4)	H45A—C45—H45C	109.5
S16—S27—S5	140.17 (5)	H45B—C45—H45C	109.5
S14—S27—S5	73.14 (4)	N6—C46—S26	123.6 (3)
S1—S27—S5	69.24 (4)	N6—C46—S25	123.5 (3)
C1—N1—C2	120.3 (4)	S26—C46—S25	112.9 (2)
C1—N1—C6	122.1 (4)	N6—C47—C48	113.0 (4)
C2—N1—C6	117.6 (3)	N6—C47—H47A	109.0
C10—N2—C15	121.1 (4)	C48—C47—H47A	109.0
C10—N2—C11A	121.6 (8)	N6—C47—H47B	109.0
C15—N2—C11A	116.6 (8)	C48—C47—H47B	109.0
C10—N2—C11B	119 (2)	H47A—C47—H47B	107.8
C15—N2—C11B	120 (2)	C49—C48—C47	114.3 (4)
C19—N3—C24A	121.5 (14)	C49—C48—C50	110.3 (5)
C19—N3—C20B	117 (3)	C47—C48—C50	106.5 (5)
C19—N3—C24B	120.6 (11)	C49—C48—H48	108.5
C20B—N3—C24B	122 (3)	C47—C48—H48	108.5
C19—N3—C20A	122.4 (11)	C50—C48—H48	108.5
C24A—N3—C20A	115.7 (18)	C48—C49—H49A	109.5
C28—N4—C29	122.2 (3)	C48—C49—H49B	109.5
C28—N4—C33	121.1 (3)	H49A—C49—H49B	109.5
C29—N4—C33	116.6 (3)	C48—C49—H49C	109.5
C37—N5—C42	120.9 (4)	H49A—C49—H49C	109.5
C37—N5—C38	120.3 (4)	H49B—C49—H49C	109.5
C42—N5—C38	118.7 (3)	C48—C50—H50A	109.5
C46—N6—C47	121.1 (3)	C48—C50—H50B	109.5
C46—N6—C51	120.8 (4)	H50A—C50—H50B	109.5
C47—N6—C51	118.0 (3)	C48—C50—H50C	109.5
N1—C1—S8	123.1 (4)	H50A—C50—H50C	109.5
N1—C1—S9	124.4 (3)	H50B—C50—H50C	109.5
S8—C1—S9	112.5 (2)	N6—C51—C52	114.1 (4)
N1—C2—C3	115.4 (4)	N6—C51—H51A	108.7
N1—C2—H2A	108.4	C52—C51—H51A	108.7
C3—C2—H2A	108.4	N6—C51—H51B	108.7
N1—C2—H2B	108.4	C52—C51—H51B	108.7
C3—C2—H2B	108.4	H51A—C51—H51B	107.6
H2A—C2—H2B	107.5	C51—C52—C53	112.4 (4)
C4—C3—C5	112.2 (6)	C51—C52—C54	108.7 (5)
C4—C3—C2	112.2 (6)	C53—C52—C54	110.8 (4)
C5—C3—C2	109.2 (5)	C51—C52—H52	108.3
C4—C3—H3	107.7	C53—C52—H52	108.3
C5—C3—H3	107.7	C54—C52—H52	108.3
C2—C3—H3	107.7	C52—C53—H53A	109.5
C3—C4—H4A	109.5	C52—C53—H53B	109.5
C3—C4—H4B	109.5	H53A—C53—H53B	109.5
H4A—C4—H4B	109.5	C52—C53—H53C	109.5
C3—C4—H4C	109.5	H53A—C53—H53C	109.5
H4A—C4—H4C	109.5	H53B—C53—H53C	109.5
H4B—C4—H4C	109.5	C52—C54—H54A	109.5
C3—C5—H5A	109.5	C52—C54—H54B	109.5
C3—C5—H5B	109.5	H54A—C54—H54B	109.5
H5A—C5—H5B	109.5	C52—C54—H54C	109.5
C3—C5—H5C	109.5	H54A—C54—H54C	109.5
H5A—C5—H5C	109.5	H54B—C54—H54C	109.5
			
C2—N1—C1—S8	−5.1 (6)	N3—C20B—C21B—C22B	−166 (4)
C6—N1—C1—S8	174.7 (3)	C19—N3—C24A—C25A	−69 (2)
C2—N1—C1—S9	173.8 (3)	C20A—N3—C24A—C25A	104 (2)
C6—N1—C1—S9	−6.3 (6)	N3—C24A—C25A—C27A	−64 (2)
Mo1—S8—C1—N1	173.1 (4)	N3—C24A—C25A—C26A	−175 (2)
Mo1—S8—C1—S9	−6.0 (2)	C19—N3—C24B—C25B	−105.8 (15)
Mo1—S9—C1—N1	−173.2 (4)	C20B—N3—C24B—C25B	68 (2)
Mo1—S9—C1—S8	5.9 (2)	N3—C24B—C25B—C27B	46 (2)
C1—N1—C2—C3	−77.1 (5)	N3—C24B—C25B—C26B	154 (2)
C6—N1—C2—C3	103.1 (5)	C29—N4—C28—S22	−179.8 (3)
N1—C2—C3—C4	−59.2 (6)	C33—N4—C28—S22	4.0 (6)
N1—C2—C3—C5	175.8 (4)	C29—N4—C28—S21	1.8 (6)
C1—N1—C6—C7	−105.5 (5)	C33—N4—C28—S21	−174.4 (3)
C2—N1—C6—C7	74.3 (6)	Mo4—S22—C28—N4	−167.1 (4)
N1—C6—C7—C8	60.6 (6)	Mo4—S22—C28—S21	11.5 (2)
N1—C6—C7—C9	−175.9 (5)	Mo4—S21—C28—N4	167.0 (4)
C15—N2—C10—S10	−177.6 (3)	Mo4—S21—C28—S22	−11.6 (2)
C11A—N2—C10—S10	−7.5 (7)	C28—N4—C29—C30	98.9 (5)
C11B—N2—C10—S10	−1.5 (13)	C33—N4—C29—C30	−84.7 (4)
C15—N2—C10—S11	2.9 (6)	N4—C29—C30—C31	−68.6 (5)
C11A—N2—C10—S11	173.0 (6)	N4—C29—C30—C32	168.4 (3)
C11B—N2—C10—S11	179.0 (13)	C28—N4—C33—C34	99.6 (4)
Mo2—S10—C10—N2	171.8 (4)	C29—N4—C33—C34	−76.8 (5)
Mo2—S10—C10—S11	−8.6 (2)	N4—C33—C34—C35	−53.0 (6)
Mo2—S11—C10—N2	−171.9 (4)	N4—C33—C34—C36	−177.1 (4)
Mo2—S11—C10—S10	8.5 (2)	C42—N5—C37—S24	4.0 (6)
C10—N2—C11A—C12A	137.0 (9)	C38—N5—C37—S24	−172.6 (3)
C15—N2—C11A—C12A	−52.5 (11)	C42—N5—C37—S23	−178.3 (3)
N2—C11A—C12A—C14A	−178.1 (8)	C38—N5—C37—S23	5.1 (6)
N2—C11A—C12A—C13A	−55.9 (14)	Mo5—S24—C37—N5	170.6 (4)
C10—N2—C11B—C12B	78 (4)	Mo5—S24—C37—S23	−7.3 (2)
C15—N2—C11B—C12B	−106 (4)	Mo5—S23—C37—N5	−170.5 (4)
N2—C11B—C12B—C14B	−165 (3)	Mo5—S23—C37—S24	7.3 (2)
N2—C11B—C12B—C13B	71 (5)	C37—N5—C38—C39	82.5 (6)
C10—N2—C15—C16	105.8 (5)	C42—N5—C38—C39	−94.1 (6)
C11A—N2—C15—C16	−64.8 (7)	N5—C38—C39—C41	68.8 (8)
C11B—N2—C15—C16	−70.3 (14)	N5—C38—C39—C40	−170.6 (7)
N2—C15—C16—C17	−54.6 (5)	C37—N5—C42—C43	80.1 (6)
N2—C15—C16—C18	−177.5 (4)	C38—N5—C42—C43	−103.3 (5)
C24A—N3—C19—S13	−16.8 (9)	N5—C42—C43—C44	66.7 (5)
C20B—N3—C19—S13	178.3 (17)	N5—C42—C43—C45	−168.9 (5)
C24B—N3—C19—S13	−7.6 (8)	C47—N6—C46—S26	173.7 (3)
C20A—N3—C19—S13	171.6 (7)	C51—N6—C46—S26	−1.2 (6)
C24A—N3—C19—S12	161.4 (8)	C47—N6—C46—S25	−4.6 (6)
C20B—N3—C19—S12	−3.5 (17)	C51—N6—C46—S25	−179.5 (3)
C24B—N3—C19—S12	170.6 (7)	Mo6—S26—C46—N6	−176.6 (4)
C20A—N3—C19—S12	−10.2 (8)	Mo6—S26—C46—S25	1.9 (2)
Mo3—S13—C19—N3	172.8 (3)	Mo6—S25—C46—N6	176.5 (4)
Mo3—S13—C19—S12	−5.61 (19)	Mo6—S25—C46—S26	−1.9 (2)
Mo3—S12—C19—N3	−172.7 (3)	C46—N6—C47—C48	−100.3 (5)
Mo3—S12—C19—S13	5.7 (2)	C51—N6—C47—C48	74.7 (5)
C19—N3—C20A—C21A	−82.5 (14)	N6—C47—C48—C49	49.7 (7)
C24A—N3—C20A—C21A	105.4 (17)	N6—C47—C48—C50	171.8 (4)
N3—C20A—C21A—C23A	−64.6 (18)	C46—N6—C51—C52	−78.5 (5)
N3—C20A—C21A—C22A	172.9 (13)	C47—N6—C51—C52	106.5 (5)
C19—N3—C20B—C21B	−113 (4)	N6—C51—C52—C53	−63.7 (5)
C24B—N3—C20B—C21B	73 (5)	N6—C51—C52—C54	173.3 (4)
N3—C20B—C21B—C23B	78 (5)		
